# Digital Gene Expression Analysis of *Populus simonii* × *P. nigra* Pollen Germination and Tube Growth

**DOI:** 10.3389/fpls.2016.00825

**Published:** 2016-06-15

**Authors:** Li-Juan Zhao, Hong-Mei Yuan, Wen-Dong Guo, Chuan-Ping Yang

**Affiliations:** ^1^State Key Laboratory of Tree Genetics and Breeding, Northeast Forestry UniversityHarbin, China; ^2^Department of Crop Molecular Breeding, Crop Breeding Institute, Heilongjiang Academy of Agricultural SciencesHarbin, China; ^3^Medical Plant Research Center, Economic Crop Institute, Heilongjiang Academy of Agricultural SciencesHarbin, China; ^4^Biotechnology Research Center, Institute of Natural Resources and Ecology, Heilongjiang Academy of SciencesHarbin, China

**Keywords:** *Populus simonii* × *P. nigra* pollen, pollen germination, pollen tube growth, transcription, DGE, differentially expressed genes

## Abstract

Pollen tubes are an ideal model for the study of cell growth and morphogenesis because of their extreme elongation without cell division; however, the genetic basis of pollen germination and tube growth remains largely unknown. Using the Illumina/Solexa digital gene expression system, we identified 13,017 genes (representing 28.3% of the unigenes on the reference genes) at three stages, including mature pollen, hydrated pollen, and pollen tubes of *Populus simonii* × *P. nigra*. Comprehensive analysis of *P. simonii* × *P. nigra* pollen revealed dynamic changes in the transcriptome during pollen germination and pollen tube growth (PTG). Gene ontology analysis of differentially expressed genes showed that genes involved in functional categories such as catalytic activity, binding, transporter activity, and enzyme regulator activity were overrepresented during pollen germination and PTG. Some highly dynamic genes involved in pollen germination and PTG were detected by clustering analysis. Genes related to some key pathways such as the mitogen-activated protein kinase signaling pathway, regulation of the actin cytoskeleton, calcium signaling, and ubiquitin-mediated proteolysis were significantly changed during pollen germination and PTG. These data provide comprehensive molecular information toward further understanding molecular mechanisms underlying pollen germination and PTG.

## Introduction

The primary function of pollen is to produce two sperm cells and transport them into the embryo sac to initiate double fertilization (Mascarenhas, 1993). In addition to their intrinsic function in sexual reproduction, the pollen tubes (PTs) have served as an ideal model for the study of cell growth and morphogenesis (Feijó et al., 2001, 2004).

Until 2002, gene-by-gene characterization had identified ~150 pollen-expressed genes from different species (reviewed in Troppmair et al., 2002), including only 23 pollen-expressed genes in *Arabidopsis thaliana*. New high-throughput technologies have enabled the analysis of male gametophyte gene expression on a global scale. SAGE (Serial analysis of gene expression) technology (Lee and Lee, 2003) and 8K Affymetrix AG microarrays (Becker et al., 2003; Honys and Twell, 2003) have allowed analysis of the pollen based on ~30% of the *Arabidopsis* genome. The application of 23K GeneChip ATH1 arrays (representing ~80% of the *Arabidopsis* genome) has enabled transcriptome analysis of pollen on a much larger scale (Pina et al., 2005). Genes in the functional categories of signaling, vesicle trafficking, cytoskeleton, and membrane transport are proportionally overrepresented in pollen (Pina et al., 2005). “Affymetrix GeneChip Rice Genome array data showed that the number of stage-enriched transcripts displays a “U-type” change during pollen development, with the lowest number at the bicellular pollen stage (Wei et al., 2010). This feature is conserved in rice (*Oryza sativa*) and *Arabidopsis* (Wei et al., 2010).” These studies revealed that mature pollen (MP) has a smaller and more unique transcriptome with a higher proportion of selectively expressed genes than vegetative tissues. Combining the three available *Arabidopsis* data resources (Honys and Twell, 2004; Zimmermann et al., 2004; Pina et al., 2005), it is estimated that the total number of genes expressed in MP is between 5000 and 7000. If the analysis is extended to cover the developmental series of pollen, the expression of ~14,000 genes is detected (Honys and Twell, 2004; Twell et al., 2006). Using Affymetrix ATH1 arrays, Borges et al. (2008) revealed that the expression of 11% of sperm cell–expressed genes is enriched in sperm cells. These sperm cell–enriched transcripts are preferentially involved in DNA repair, ubiquitin-mediated proteolysis, and cell cycle progression. The high levels of expression of ubiquitin pathway–related genes in generative cells suggests that ubiquitin proteolysis plays a critical role in the male gametogenesis of higher plants (Singh and Bhalla, 2007). Although greater emphasis has been put on model systems such as *Arabidopsis*, there is still a place for non-model systems in these studies.

In contrast to the abundance of molecular information available about pollen development and maturation, little is known about the genome-wide events underlying pollen germination (PG) and pollen tube growth (PTG), which is essential for understanding the molecular mechanisms of polar tube growth and invasion into pistils (Wei et al., 2010). Many low-abundance transcripts cannot be analyzed by microarrays (McCormick, 2004). Guyon et al. (2000) identified ~20 cDNAs related to PG, many of which corresponded to low-abundance mRNAs. Thus, it is difficult to identify genes that are transcribed only upon PG or to identify mRNAs that are essentially not translated until the end of PG (Wittink et al., 2000). However, Solexa deep-sequencing approaches overcome many of the inherent limitations of traditional sequencing systems and thus make it possible to detect low-abundance transcripts and alternative splicing events. For *Arabidopsis*, Wang et al. (2008) reported that, compared with MP, hydrated pollen (HP) and PTs have a larger number of newly transcribed genes. Inhibition experiments demonstrated that PG and polar tube growth strictly depend on protein synthesis but are relatively independent of transcription in many plant species (Mascarenhas, 1993; Honys and Twell, 2004; Wang et al., 2004; Hao et al., 2005). Thus, *de novo* synthesis of transcripts in germinating pollen might not be crucial for PG and early tube growth, although this issue needs to be addressed by sequential comparison of transcriptomes between developing and germinated pollen (Wei et al., 2010). To investigate the transcriptome changes of pollinated pollen tubes, a semi—*in vivo* approach was used, identifying 383 genes enriched in pollen tubes emerging from cut pistils compared with *in vitro*—cultured ones (Qin et al., 2009). Recently, a study by Lin et al. (2014) revealed over 500 transcripts specifically enriched in *in vivo*–grown pollen tubes. Surprisingly, there was only one gene overlap in these two studies.

*Populus simonii* × *P. nigra* is widely cultivated in the plains of Heilongjiang province in China and has many useful features such as cold and drought resistance, relatively high salinity tolerance, and fast growth. *P. simonii* × *P. nigra* pollen is easily accessible and has longevity under *in vitro* conditions. The MP grain is bicellular. Until recently, the bicellular pollen transcriptome of only two plants, soybean (*Glycine max*) and tobacco (*Nicotiana tabacum* cv. Samsun), had been analyzed (Haerizadeh et al., 2009; Hafidh et al., 2012). Our present study is the first study to integrate gene expression analysis for poplar pollen and PTs. To better understand the molecular regulation mechanism of PG and rapid tube growth, we constructed three libraries using the Illumina Solexa digital gene expression (DGE) system and compared the gene expression profiles at three developmental stages of *P. simonii* × *P. nigra* pollen. We also discovered a large number of differentially expressed genes (DEGs) involved in crucial functional categories and pathways during PG and PTG. These genes may play important roles in regulating PG and PTG and thus are attractive candidates for further analysis. These results also lay the theoretical foundation for tree crossbreeding and overcoming self-incompatibility.

## Materials and methods

### Plant material

Budding male-flower branches of *P. simonii* × *P. nigra* were collected from five different trees growing on the Northeast Forestry University campus. The branches were cultured in water at room temperature (17 ± 1°C). MP grains were obtained after 5 days. These pollen grains were dried overnight at room temperature and filtered using a stainless steel mesh (50 μm). The desiccated MP grains were stored at −80°C until use. The purity of MP was determined under a light microscope using 4′,6-diamimophenylindole (DAPI) staining.

### *In vitro* PG and collection of HP and PT

*P. simonii* × *P. nigra* pollen grains stored at −80°C were resuscitated at 5°C for 2 h, and 10 mg of the resuscitated pollen was evenly scattered in 10 mL liquid medium using a stainless steel mesh (50 μm). The liquid medium for PG and PTG consisted of 15% sucrose, 40 mg L^−1^ CaCl_2_, 20 mg L^−1^H_3_BO_3_ (pH 6.0–6.3). Pollen grains were incubated in a 100 mL flask at 21°C in darkness for ~7 h, which resulted in ~75% germination and an average PT length of ~250 μm. The PTs were filtered using a 50-μm nylon mesh to remove the ungerminated pollen grains and collected from the nylon mesh for total RNA extraction. HP samples were collected and centrifuged at 27 **(**× *g*) rcf after resuscitated pollen grains were incubated for 1.5 h. HP viability was assessed with FDA staining. Resuscitated pollen grains were incubated for 1.5 h in liquid medium with 2 μg mL^−1^ fluorescein diacetate (FDA). After washing out the FDA with liquid medium, the fluorescence of pollen grains was observed using a Zeiss SteREO Lumar. V12 fluorescence microscope.

### RNA extraction, tag library construction, and sequencing

Total RNA was isolated separately from the three samples (MP, HP, PT) using Trizol Reagent (Invitrogen, USA) and treated with DNase I (Fermentas, USA). RNA quality and purity were assessed with the OD 260:230 ratio and RNA integrity number using a 2100 Bioanalyzer (Agilent Technologies, Boblingen, Germany). To obtain comprehensive gene expression information, the pooled RNA from the MP, HP, and PT samples from five different trees were used for expression profiles analysis.

Next, a DGE library was prepared using the Digital Gene Expression Tag Profile kit (Illumina). The detailed procedure referred Sun et al.'s method (2012). Sequencing was performed by HuaDa Gene (http://www.genomics.cn/index) using the sequencing-by-synthesis method to produce the raw tag data. Raw data (MP, HP, and PT) have been deposited in Sequence Read Archive (SRA) of NCBI and can be accessed from (http://trace.ncbi.nlm.nih.gov/Traces/sra_sub/sub.cgi?subid=605060&from=list&action=show:submission">http://trace.ncbi.nlm.nih.gov/Traces/sra_sub/sub.cgi?subid=605060&from=list&action=show:submission">http://trace.ncbi.nlm.nih.gov/Traces/sra_sub/sub.cgi?subid=605060&from=list&action=show:submission) using the accession number PRJNA312293.

### Analysis of sequencing data and tag mapping

We sequenced the DGE libraries for stages MP, HP, and PT using the Illumina massively parallel sequencing platform. Raw sequences were filtered as described (Sun et al., 2012). A preprocessed database of all possible CATG+17-nt tag sequences was created using *Populus trichocarpa* (http://genome.jgi-psf.org/Poptr1_1/Poptr1_1) as a reference gene sequence. To monitor the mapping of the two strands, both the sense and the complementary antisense sequences were included in the data collection. All clean tags were mapped to the reference sequence, with = 1 nt mismatch allowed. Clean tags mapped to the reference sequence from multiple genes were excluded. The remaining clean tags were considered unambiguous clean tags and were annotated. The number of unambiguous clean tags for each gene was calculated and then normalized to the total number of transcripts per million clean tags (t Hoen et al., 2008; Morrissy et al., 2009). A gene detected only in one stage (MP, HP, or PT) was defined as a specifically expressed gene in that stage.

### Analysis of DEGs

DEGs between two samples were identified according to the method of Audic and Claverie (1997). *P*-value was used to test differential transcript accumulation. False Discovery Rate (FDR) was used to determine the threshold *P*-value in multiple testing and analysis. Assuming that R differentially expressed genes have been selected, S genes really show differential expression, whereas the other V genes are false positives. If the error ratio “Q = V/R” must remain below a cutoff (5%), FDR should not exceed 0.05 (Benjamini and Yekutieli, 2001). To gain the global transcriptional changes that were associated with PG and PTG and to assess the molecular basis of PG and PTG, we used FDR ≤ 0.001, *P* < 0.0005, and ∣log2 ratio∣ ≥ 1 as the threshold to judge the significance of gene expression difference. The DEGs were then subjected to gene ontology (GO) and Kyoto Encyclopedia of Genes and Genomes (KEGG) ontology enrichment analysis. Enriched *P*-values were calculated according to the hypergeometric test of Sun et al. (2012). A corrected *P* < 0.05 was selected as the threshold for significant enrichment of the gene sets. Annotations of the GO and pathways of the reference sequences were obtained using BLAST v2.2.21 software (ftp://ftp.ncbi.nlm.nih.gov/blast/executables/blast+/2.2.21/). Cluster analysis of gene expression patterns was performed with Cluster (Eisen et al., 1998) and Java Treeview software (Saldanha, 2004). We performed the cluster analysis of the intersection (expressed in the three stages and corresponding FDR ≤ 0.001 and |log2 ratio|≥ 1) of DEGs.

### Quantitative reverse transcription (qRT)-PCR

We used qRT-PCR to verify the DGE results. The RNA samples (MP, PT) used for qRT-PCR were the same as those for the DGE experiments. Primers were designed using Primer Premier 5.0 (Premier, Canada). Actin, 18S rDNA, and GAPDH were used as the internal controls to normalize the data. The PCR primers are shown in Supplementary Table S1. The qRT-PCR was performed using the DNA Engine Opticon TM2 fluorescence detection system **(**MJ Research, USA) with SYBR-Green (SYBR PrimeScript RT-PCR kit, TaKaRa Biotechnology Co., Ltd., Dalian, China). Each reaction (25 μL) contained 0.5 μL of each primer, 2 μL cDNA, 12.5 μL SYBR Premix Ex TaqTM, and 9.5 μL double-distilled H_2_O. All runs included a negative control without template cDNA that produced no detectable fluorescence signal. The amplification reactions were initiated with pre-denaturing at 94°C for 30 s, followed by 45 cycles of denaturing (94°C for 12 s), annealing (54°C for 30 s), and extension (72°C for 45 s; 78.5°C for 1 s), and a final step of 55–99°C to plot a dissociation curve for each amplified product. All reactions were performed with at least three replicates. Relative expression was calculated with the 2^−ΔΔCt^ method (Livak and Schmittgen, 2001).

## Results

### Cytological observation and in vitro germination of *P. simonii* × *P. nigra* pollen

**Figure 1:**
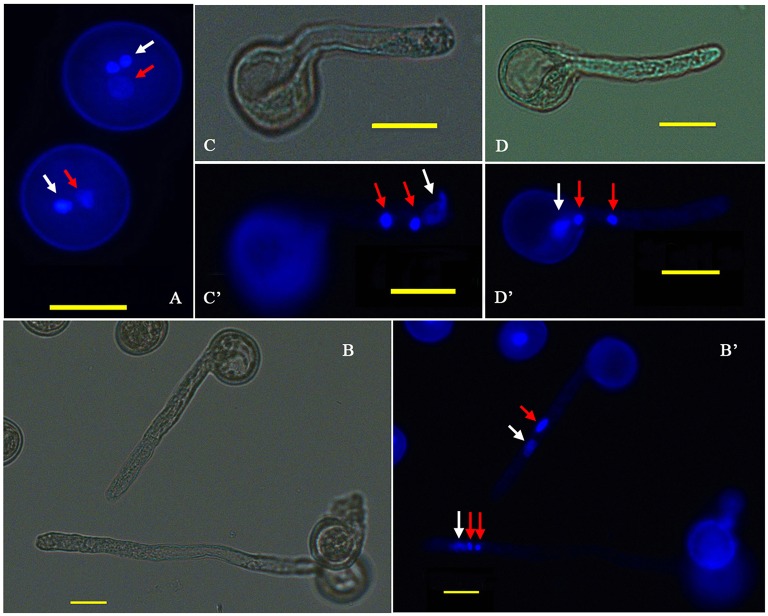
**Cytological observation of *P. simonii* × *P. nigra* PG and PTG. (A)** DAPI fluorescence image of two-celled and three-celled pollen. **(B,B**′**)** Bright-field and DAPI fluorescence images of the two and three nuclei states in the tube; red arrows show generative nuclei or two sperm nuclei, and white arrows show vegetative nuclei. **(C,C**′**)** Bright-field and DAPI fluorescence images, respectively, of the vegetative nucleus leading the sperm nucleus into the PT. **(D,D**′**)** Bright-field and DAPI fluorescence images, respectively, of two sperm nuclei moving into the tube before the vegetative nucleus.

**Figure 2:**
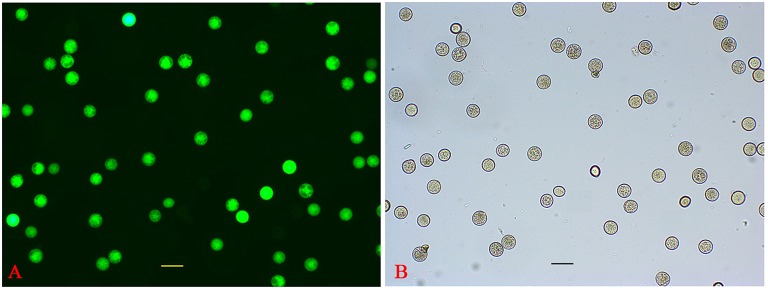
**Evaluation of HP viability. (A)** FDA fluorescence image of HP. **(B)** Bright-field image of HP.

*P. simonii* × *P. nigra* MP grains were stained with DAPI and examined by fluorescence microscopy. The vast majority of them were bicellular, consisting of a large vegetative cell and a small generative cell. A very small proportion of tricellular pollen (containing one large vegetative cell and two sperm cells) was also observed (Figure 1A). Of the >1500 pollen grains counted, tricellular pollen accounted for only ~0.089%. The germinating pollen could transport an entire germ unit into the pollen tube (Figures 1B,B′). For the bicellular pollen, the vegetative nucleus was transferred into the pollen tube first, followed by the generative cell, and then the generative cell was divided into two sperm cells in the pollen tube. For the tricellular pollen, the two sperm cells were transferred into the pollen tube first, followed by the vegetative nucleus (Figures 1C,C′,D,D′). It was not clear why the tricellular pollen appeared in our experiments. The germination rate was ~75% when the pollen was cultured in the liquid medium for 7 h >90% of the HP grains were positive for FDA staining, indicating high-level viability of the cultured pollen grains (Figure 2). We proposed that the pollen grains might enter the germination process along with the emergence of the pollen tube. Given that transcriptional changes can be extremely rapid, they should appear before any morphological change is observed (Wang et al., 2008). Approximately 15% of HP grains showed emergence of PTs, suggesting that RNA extracted at this stage would provide transcriptional information about PG.

### Analysis of the digital gene expression libraries

The major characteristics of three libraries are summarized in Table 1. We obtained ~3.5 million total sequence tags per library, with 281,827 distinct tag sequences. We filtered raw sequence, leaving ~3.3 million total clean sequence tags per library, 105,295 of which were distinct. The MP library showed the highest numbers for both total and distinct sequence tags, followed by the HP and the PT libraries. Moreover, the MP library exhibited the highest percentage of distinct clean, high-copy-number tags and the lowest ratio of distinct to total tags. As shown in Table 1, the read copy numbers ranged from 2 to ≥100. The majority of the distinct clean tags (~63% from each library) had low copy number (< 20 copies), and ~22% of the tags from each library had a copy number between 11 and 100. Only ~4% of the tags were detected >100 times.

**Table 1:** **Major characteristics of DGE libraries and tag mapping to reference sequences database**.

	**MP**		**HP**		**PT**	
	**Distinct tag**	**Total tag number**	**Distinct tag**	**Total tag number**	**Distinct tag**	**Total tag number**
Raw data	346,420	4,746,710	286,685	3,188,914	212,377	2,637,062
Tags containing N	39,861	57,487	26,579	37,599	23,156	32,326
Adaptors	0	0	0	0	0	0
Tag CopyNum < 2	180,056	180,056	161,267	161,267	98,679	98,679
Clean tag	126,503	4,509,167	98,839	2,990,048	90,542	2,506,057
CopyNum > = 2	126,503	4,509,167	98,839	2,990,048	90,542	2,506,057
CopyNum >5	51,791	4,297,810	36,024	2,816,071	34,573	2,342,951
CopyNum >10	33,568	4,159,257	22,702	2,715,125	20,722	2,238,622
CopyNum >20	20,945	3,974,730	13,751	2,584,585	12,131	2,113,464
CopyNum >50	10,372	3,637,058	6569	2,356,948	5621	1,907,976
CopyNum >100	5733	3,309,786	3719	2,155,101	3012	1,724,226
Tag mapping						
All mapping	59,339	2,340,206	41,979	1,381,560	39,734	1,215,984
Unambiguous mapping	47,294	1,659,654	32,998	968,992	31,259	824,530
Mapping to genome	23,473	990,689	17,076	705,316	15,666	493,927
Unknown tag	24,469	561,809	23,781	442,972	21,682	463,703

*All Mapping represents all Tags Mapping to Gene (Sense and Anti-Senese). Unambiguous Mapping represents unambiguous Tag Mapping to Gene (Sense and Anti-Sense). Mapping to genome represents the clean tags that can not be mapped to mRNA, mitochondria and chloroplast are mapped to the whole genome, providing zones that can be uniquely mapped by those tags*.

Saturation analysis of the libraries showed that the number of newly emerging distinct tags gradually decreased with the increase in total sequence tags. Furthermore, when the number of sequencing tags reached 2.0, 1.5, and 1.0 million for the MP, HP, and PT libraries, respectively, the libraries became saturated (Supplementary Figure S1).

### Analysis of tag mapping

For reference gene sequences from *P. trichocarpa*, altogether, 41,404 genes (90.1%) had the CATG site, resulting in a total of 1,192,383 reference tags with 167,529 (81.1%) unambiguous tags. In the present study, we were able to map ~70.6% of the total clean tags to genes or genome positions, demonstrating the feasibility of using this approach to investigate PG and PTG in *P. simonii* × *P. nigra*. 46.9, 42.5, and 43.9% of the distinct clean tags in the MP, HP and PT libraries, respectively, mapped to the reference tag database, 37.4, 33.4, and 34.5% of the distinct clean tags of the three libraries mapped unambiguously to the unigenes, and 18.6, 17.3, and 17.3% of the distinct clean tags matched genomic sequences within each library (Table 1). The percentage of total clean tags that matched to the genome was the highest for HP (23.6%). As a result of the considerable sequencing depth of Illumina/Solexa technology and incomplete annotation of the *P. trichocarpa* genome, 19.3, 24.1, and 24.0% of the tags in the MP, HP, and PT libraries were unmatched (Table 1). It perhaps indicates that most of the genes involved in PG and PTG had not yet been identified. But these collections of tags will be highly useful as more *P. trichocarpa* genomic sequences become available.

In addition, ~16% of the distinct tags in the three libraries mapped to the antisense strand, suggesting that these regions might be bi-directionally transcribed (Supplementary Table S2). Approximately 2421 antisense-stand transcripts were detected in each library, and the ratio of sense to antisense strand transcripts was ~4.2:1 for all libraries (Supplementary Table S3). This result suggested that the transcriptional regulation during PG and PTG is mostly directed at the sense strand.

### Genes expressed in MP, HP, and PT

More genes were detected in the MP library than the HP and PT libraries. Specifically, 11,751 (25.6% of reference genes), 9348 (20.3%), and 9644 (21.0%) unambiguous tag-mapped genes were detected in MP, HP, and PT, respectively (Table 2). In total, 13,017 genes (representing 28.3% of the unigenes in the reference) were present in at least one of MP, HP, or PT (Table 2, Supplementary Table S4). The numbers of transcripts detected were higher than reported in previous microarray-based studies (Honys and Twell, 2004; Pina et al., 2005; Wang et al., 2008). Among these genes, 6996 (15.2%) were consistently expressed in all three stages (Table 2, Supplementary Table S5), and another 6021 genes were preferentially expressed in one or two stages. Further analysis of these 6021 genes showed that 2006 were specifically expressed in MP (17.1% of the expressed genes in MP), 450 were specifically expressed in HP (4.8% of the expressed genes in HP), and 958 were specifically expressed in PT (9.9% of the expressed genes in PT; Table 2).

**Table 2:** **Numbers of expressed genes, specifically expressed genes, and transcriptionally changed genes during PG and PTG**.

**Gene category**	**No. of Genes**	**Percentage**
Genes expressed in		Percentage in Ref genes (41,404 genes)
MP	11,751	28.38
HP	9348	22.58
PT	9644	23.29
All three stages	6996	16.90
At least one stage	13,017	31.44
Genes specifically expressed in		Percentage in respective stage
MP	2006	17.07
HP	450	4.81
PT	958	9.93
Trancriptionally changed genes		Percentage in total expressed genes (13,017genes)
Up-regulated in PG	128	0.98
Down-regulated in PG	990	7.61
Up-regulated in PTG	741	5.69
Down-regulated in PTG	609	4.68

*The first column indicates three stages of MP, HP, PT, and two process of PG and PTG. The second and the third column indicates the numbers of expressed genes, specifically expressed genes, and transcriptionally changed genes and the relative percentages*.

### Identification of DEGs and GO analysis during PG and PTG

**Figure 3:**
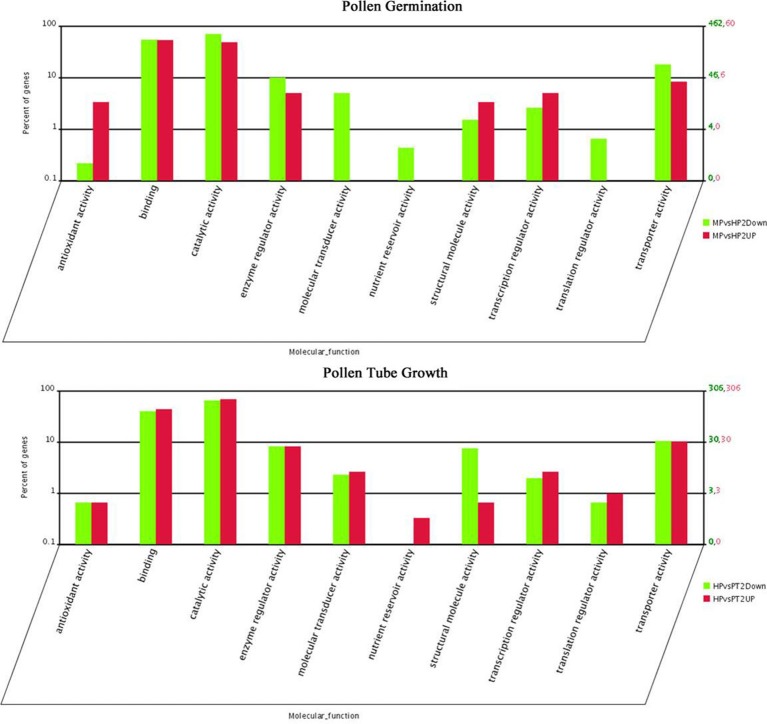
**Functional category distribution of DEGs in PG and PTG**. MPvsHP2UP and HPvsPT2UP indicate genes showing ≥two-fold increase in expression during PG and PTG; MPvsHP2Down and HPvsPT2Down indicate genes showing a ≥two-fold decrease in expression during PG and PTG.

The distribution of fold-changes in tag number among the three libraries is shown in Supplementary Figure S2. The great majority of transcripts were expressed at similar levels in the three libraries: approximately 96.7% tags showed a < five-fold difference in expression. 1118 and 1350 DEGs were identified during PG and PTG, respectively. There were 128 up-regulated and 990 down-regulated transcripts during PG and 741 up-regulated and 609 down-regulated transcripts during PTG (Table 2, Supplementary Table S6). Except for the unknown transcripts (~54%), predicted or known genes were categorized according to their functions, cellular component, and biological process. Altogether, 60 up-regulated and 462 down-regulated genes during PG and 306 up-regulated and 306 down-regulated genes during PTG were classified into 10 main functional categories (Figure 3, Supplementary Table S7). The categories, catalytic activity, binding, transporter activity, and enzyme regulator activity were overrepresented during PG and PTG. The percentage of up-regulated genes in the categories of antioxidant activity, structural molecule activity, molecular transducer activity, nutrient reservoir activity, and translation regulator activity were greatly different during PG and PTG (Figure 3). In PG, Go analysis showed that 25 terms (GO cellular component) were significantly enriched, including those asscociated with protein complex, endomembrane system, membrane part, vesicle membrane, and cytoplasmic membrane-bounded vesicle (Supplementary Table S8). Significantly enriched “cellular component” was not found during PTG. Eight terms (GO biological process) included those associated with transport, establishment of localization, localization, ion transport, and cation transport were significantly enriched during PG (Supplementary Table S8). But we identified three terms (GO biological process) different from PG, including those associated with sphingolipid metabolic process, membrane lipid metabolic process and alcohol metabolic process during PTG (Supplementary Table S8).

### Clustering analysis of DEGs

**Figure 4:**
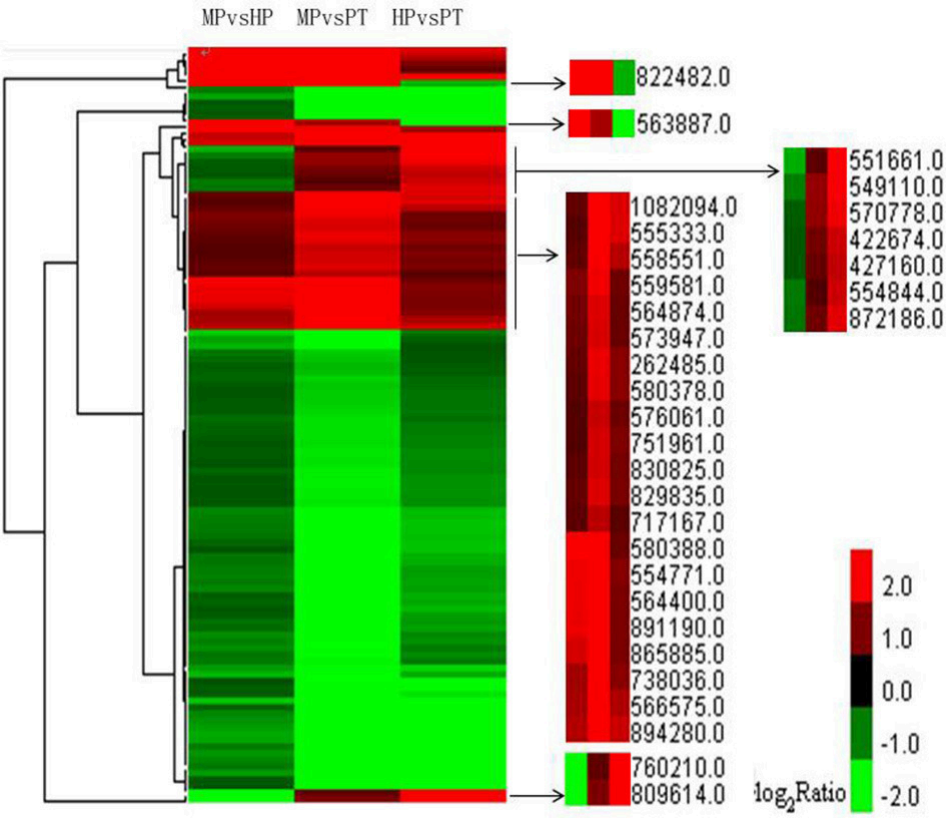
**Clustering analysis of DEGs from the three stages with FDR ≤ 0.001 and |log2 ratio| ≥ 1 within each group of DEGs**. Altogether 115 genes were used in the clustering analysis. Red represents up-regulated expression; green represents down-regulated expression.

Clustering analysis showed that some highly dynamic genes were detected. Two genes—one involved in peroxidase activity (POPTRDRAFT_822482) and the other in ATP binding (POPTRDRAFT_563887)—were significantly up-regulated during PG but markedly down-regulated during PTG. A gene of unknown function (POPTRDRAFT_760210) and a gene involved in protein kinase activity (POPTRDRAFT_809614) were significantly up-regulated during PTG but significantly down-regulated during PG (Figure 4). Another seven genes followed a similar trend. Among these are genes involved in the microtubule-associated complex or microtubule motor activity (POPTRDRAFT_551661), catalytic activity (POPTRDRAFT_570778), and the ubiquitin ligase complex or protein ubiquitination (POPTRDRAFT_554844). The molecular functions of the other four genes (POPTRDRAFT_549110, POPTRDRAFT_422674, POPTRDRAFT_427160, and POPT RDRAFT_872186) remain unknown (Figure 4). The DEG clustering analysis also showed that some genes were up-regulated during both PG and PTG, including genes involved in pectinesterase activity (POPTRDRAFT_555333), membrane or transport activity (POPTRDRAFT_262485, POPTRDRAFT_751961, and POPTRDRAF T_830825), regulation of transcription (POPTRDRAFT_564400), protein nuclear import (POPTRDRAFT_ 554771), protein modification (POPTRDRAFT_738036), and calcium ion binding (POPTRDRAFT_576061; Figure 4). The functions of more than half of the genes that were up-regulated during PG and PTG remain unknown.

### Pathway enrichment analysis of DEGs

There were 620 and 728 DEGs with pathway annotations during PG and PTG, respectively. A total of 186 pathways were enriched during PG (Supplementary Table S9), the first 43 of which are significantly enriched (Table 3). There were 207 enriched pathways during PTG (Supplementary Table S10), the first 24 of which are significantly enriched (Table 4).

**Table 3:** **Significantly enriched pathways during PG(*Q*-value < 0.05)**.

	**Pathway**	**DEGs with pathway annotation (620)**	**All genes with pathway annotation (22,673)**	***P*-value**	***Q*-value**	**KEGG Pathway ID**
1	ErbB signaling pathway	13 (2.1%)	85 (0.37%)	5.13E-07	9.35686E-05	ko04012
2	TGF-β signaling pathway	13 (2.1%)	90 (0.4%)	1.01E-06	9.35686E-05	ko04350
3	mTOR signaling pathway	13 (2.1%)	103 (0.45%)	4.74E-06	0.00029372	ko04150
4	Dorso-ventral axis formation	11 (1.77%)	85 (0.37%)	2.00E-05	0.000822388	ko04320
5	Natural killer cell mediated cytotoxicity	11 (1.77%)	86 (0.38%)	2.23E-05	0.000822388	ko04650
6	MAPK signaling pathway	22 (3.55%)	299 (1.32%)	2.84E-05	0.000822388	ko04010
7	Long-term depression	11 (1.77%)	89 (0.39%)	3.10E-05	0.000822388	ko04730
8	Focal adhesion	17 (2.74%)	203 (0.9%)	4.62E-05	0.000942361	ko04510
9	T cell receptor signaling pathway	11 (1.77%)	93 (0.41%)	4.68E-05	0.000942361	ko04660
10	GnRH signaling pathway	16 (2.58%)	185 (0.82%)	5.21E-05	0.000942361	ko04912
11	MAPK signaling pathway–fly	7 (1.13%)	37 (0.16%)	5.57E-05	0.000942361	ko04013
12	Non-small cell lung cancer	10 (1.61%)	80 (0.35%)	6.40E-05	0.000992421	ko05223
13	Renal cell carcinoma	10 (1.61%)	84 (0.37%)	9.74E-05	0.001393244	ko05211
14	Acute myeloid leukemia	9 (1.45%)	70 (0.31%)	0.000118662	0.001576505	ko05221
15	Pathways in cancer	20 (3.23%)	290 (1.28%)	0.000156568	0.001941443	ko05200
16	Thyroid cancer	8 (1.29%)	58 (0.26%)	0.000171678	0.001995759	ko05216
17	Prostate cancer	13 (2.1%)	147 (0.65%)	0.000206339	0.002257594	ko05215
18	Gap junction	11 (1.77%)	113 (0.5%)	0.000273245	0.002823531	ko04540
19	VEGF signaling pathway	11 (1.77%)	114 (0.5%)	0.000295107	0.002888943	ko04370
20	Insulin signaling pathway	19 (3.06%)	294 (1.3%)	0.000509545	0.004738766	ko04910
21	Phosphatidylinositol signaling system	17 (2.74%)	253 (1.12%)	0.000639121	0.005660788	ko04070
22	Long-term potentiation	14 (2.26%)	188 (0.83%)	0.000695286	0.005878324	ko04720
23	Tight junction	18 (2.9%)	285 (1.26%)	0.000922378	0.007459228	ko04530
24	Type II diabetes mellitus	7 (1.13%)	62 (0.27%)	0.001477238	0.011448595	ko04930
25	Chemokine signaling pathway	9 (1.45%)	101 (0.45%)	0.001805983	0.013436514	ko04062
26	Melanogenesis	13 (2.1%)	189 (0.83%)	0.002173863	0.015551482	ko04916
27	Sphingolipid metabolism	8 (1.29%)	85 (0.37%)	0.002266143	0.015611207	ko00600
28	Fc ε RI signaling pathway	7 (1.13%)	70 (0.31%)	0.002986459	0.019101208	ko04664
29	B cell receptor signaling pathway	8 (1.29%)	89 (0.39%)	0.003025235	0.019101208	ko04662
30	Regulation of actin cytoskeleton	15 (2.42%)	244 (1.08%)	0.00308084	0.019101208	ko04810
31	Endometrial cancer	8 (1.29%)	92 (0.41%)	0.003714169	0.022285014	ko05213
32	Inositol phosphate metabolism	12 (1.94%)	179 (0.79%)	0.003895542	0.022642838	ko00562
33	Glioma	12 (1.94%)	182 (0.8%)	0.004448137	0.025071318	ko05214
34	Chronic myeloid leukemia	7 (1.13%)	76 (0.34%)	0.004735616	0.025906605	ko05220
35	Axon guidance	8 (1.29%)	98 (0.43%)	0.005450439	0.028160602	ko04360
36	Colorectal cancer	8 (1.29%)	98 (0.43%)	0.005450439	0.028160602	ko05210
37	Wnt signaling pathway	13 (2.1%)	215 (0.95%)	0.006467202	0.031924369	ko04310
38	Adherens junction	8 (1.29%)	101 (0.45%)	0.006522183	0.031924369	ko04520
39	Vascular smooth muscle contraction	12 (1.94%)	192 (0.85%)	0.006757216	0.032226723	ko04270
40	Melanoma	7 (1.13%)	82 (0.36%)	0.007163473	0.032497707	ko05218
41	Bladder cancer	7 (1.13%)	82 (0.36%)	0.007163473	0.032497707	ko05219
42	Vibrio cholerae infection	8 (1.29%)	105 (0.46%)	0.008190482	0.036144692	ko05110
43	Progesterone-mediated oocyte maturation	10 (1.61%)	150 (0.66%)	0.008356031	0.036144692	ko04914

**Table 4:** **Significantly enriched pathways during PTG (*Q*-value < 0.05)**.

	**Pathway**	**DEGs with pathway annotation (728)**	**All genes with pathway annotation (22,673)**	***P*-value**	***Q*-value**	**Pathway ID**
1	Alzheimer's disease	28 (3.85%)	350 (1.54%)	9.95E-06	0.001746525	ko05010
2	Sphingolipid metabolism	12 (1.65%)	85 (0.37%)	1.69E-05	0.001746525	ko00600
3	Galactose metabolism	17 (2.34%)	173 (0.76%)	4.46E-05	0.002505594	ko00052
4	SNARE interactions in vesicular transport	11 (1.51%)	80 (0.35%)	4.84E-05	0.002505594	ko04130
5	Epithelial cell signaling in Helicobacter pylori infection	10 (1.37%)	75 (0.33%)	0.000137991	0.005712832	ko05120
6	Parkinson's disease	20 (2.75%)	263 (1.16%)	0.00034861	0.012027028	ko05012
7	Huntington's disease	26 (3.57%)	394 (1.74%)	0.000461303	0.01364138	ko05016
8	Pathogenic Escherichia coli infection –EPEC	8 (1.1%)	63 (0.28%)	0.000885697	0.020371026	ko05131
9	Pathogenic Escherichia coli infection–EHEC	8 (1.1%)	63 (0.28%)	0.000885697	0.020371026	ko05130
10	Gap junction	11 (1.51%)	113 (0.5%)	0.001035227	0.021429199	ko04540
11	Phosphatidylinositol signaling system	18 (2.47%)	253 (1.12%)	0.001444927	0.027190899	ko04070
12	ErbB signaling pathway	9 (1.24%)	85 (0.37%)	0.001614039	0.027842173	ko04012
13	Inositol phosphate metabolism	14 (1.92%)	179 (0.79%)	0.001977064	0.030086932	ko00562
14	Vibrio cholerae infection	10 (1.37%)	105 (0.46%)	0.002034865	0.030086932	ko05110
15	Calcium signaling pathway	15 (2.06%)	204 (0.9%)	0.002539515	0.032077558	ko04020
16	Glycolysis / Gluconeogenesis	19 (2.61%)	290 (1.28%)	0.002767816	0.032077558	ko00010
17	Prostate cancer	12 (1.65%)	147 (0.65%)	0.002841059	0.032077558	ko05215
18	Glycerophospholipid metabolism	19 (2.61%)	292 (1.29%)	0.002985572	0.032077558	ko00564
19	Long-term potentiation	14 (1.92%)	188 (0.83%)	0.003102072	0.032077558	ko04720
20	Oxidative phosphorylation	23 (3.16%)	383 (1.69%)	0.003213484	0.032077558	ko00190
21	Melanogenesis	14 (1.92%)	189 (0.83%)	0.003254245	0.032077558	ko04916
22	Ubiquitin-mediated proteolysis	25 (3.43%)	435 (1.92%)	0.003869745	0.036410783	ko04120
23	Wnt signaling pathway	15 (2.06%)	215 (0.95%)	0.004163601	0.036878991	ko04310
24	Axon guidance	9 (1.24%)	98 (0.43%)	0.004275825	0.036878991	ko04360

The first 43 significantly enriched pathways during PG included some critical pathways, such as the mitogen-activated protein kinase (MAPK) signaling pathway, the phosphatidylinositol signaling system, sphingolipid metabolism, regulation of the actin cytoskeleton, and inositol phosphate metabolism (Table 3). In PG, the top three most enriched pathways were the ErbB, TGF-β, and mTOR signaling pathways often associated with various cancers (Modjtahedi et al., 2014; Azad et al., 2015). This group also included other pathways such as the gap, tight and adherens junctions pathways, insulin signaling pathways, and a pathway associated with type II diabetes mellitus (Table 3). Although most of the latter pathways have been well studied in animals and humans, their functions in plants remain unclear. The first 24 significantly enriched pathways during PTG mainly included sphingolipid metabolism, galactose metabolism, SNARE interactions in vesicular transport, the calcium signaling pathway, the phosphatidylinositol signaling system, inositol phosphate metabolism, glycerophospholipid metabolism, oxidative phosphorylation, ubiquitin-mediated proteolysis, and some pathways related to human diseases (Table 4).

### qRT-PCR confirmation

**Figure 5:**
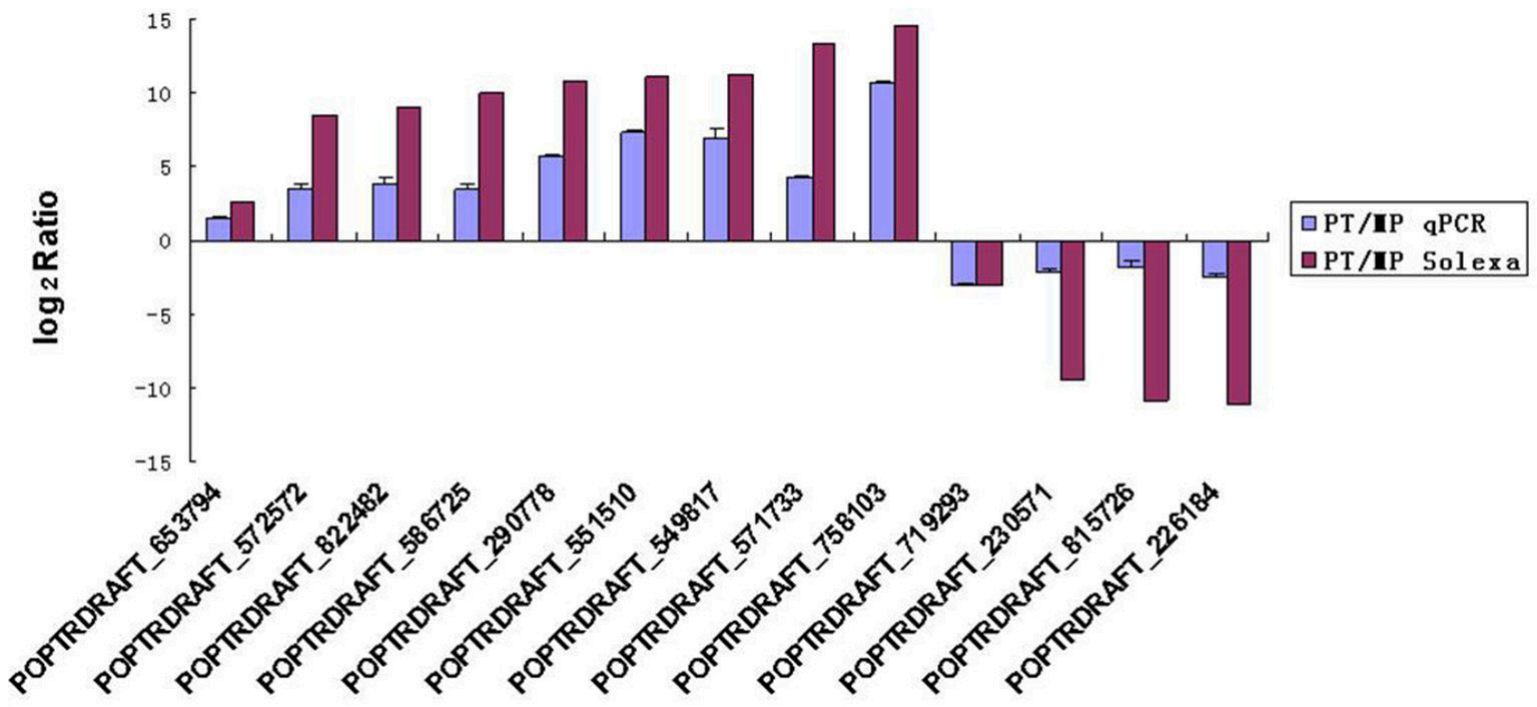
**qRT-PCR validation of Solax digital gene expression data**. PT:MP ratios of 13 tested genes determined by Illumina/Solexa compared with ratios determined by qRT-PCR. The expression profile trends of the two methods were similar. Error bars represent SE.

To evaluate the validity of the Illumina/Solexa analysis and to further assess the patterns of differential gene expression, 13 candidate genes were selected from the two developmental stages used for Illumina/Solexa analysis (MP and PT) and examined by qRT-PCR. Of these genes, nine were up-regulated and four were down-regulated (Figure 5). The expression profile trends of the qRT-PCR for these selected genes were similar to those detected by the Illumina/Solexa sequencing–based method, further confirming the Illumina/Solexa data.

## Discussion

### Transcriptional level analysis during PG and PTG in *P. simonii* × *P. nigra*

Our data showed that the total number and diversity of transcribed genes greatly decreased from MP to HP and slightly increased from HP to PT. Stage-specifically expressed genes followed the same trend. Down-regulated genes were dominant during PG, whereas up-regulated genes were dominant during PTG. These results suggest a shift in the gene expression program in PG and PTG and that the HP stage may be crucial for the regulation of this shift. Wang et al. (2008) however, reported that the numbers of transcribed and specifically expressed genes gradually increase from MP to HP and further to PT, and that up-regulated genes are dominant during PG and PTG compared with down-regulated genes in *Arabidopsis*. Similar results have been seen in tobacco gene expression (Hafidh et al., 2012), but that study only compared two time points (MP grains and *in vitro*–germinated PTs grown for 4 h), and the gene expression differences between the two points are marginal. The contradiction between our results and these previous two studies might be due to differences in species, sample preparation, or sequencing platforms. *P. simonii* × *P. nigra* and tobacco have bicellular pollen, whereas *Arabidopsis* has tricellular pollen. As shown in several plant studies, PG and early PTG are not dependent on new RNA synthesis. In most plants that shed bicellular pollen, the later PTG and division of the generative cell into two sperm cells are dependent on new mRNA synthesis after the pollen tube forms (reviewed in Mascarenhas, 1975). During the first hour of PTG, ~50% of the protein synthesis utilizes previously existing mRNAs, and the remaining 50% utilizes newly synthesized mRNAs (Mascarenhas and Mermelstein, 1981). These studies are consistent with our findings. Our results showed that the proportion of specifically expressed genes and the number of up-regulated genes significantly increased from HP to PT (Table 2), suggesting that these genes might play critical roles during PTG and sperm cell formation.

### Functional categories and genes involved in PG and PTG

The up-regulated genes involved in antioxidant, structural molecule, and transcription regulator activity were dominant during PG compared with the down-regulated genes. And the percentage of up-regulated genes involved in antioxidant activity and structural molecule activity categories was significantly higher than PTG (Figure 3, Supplementary Table S7). Reactive oxygen species (ROS) played a key role in pollen grain activation and pollen tube initiation in kiwifruit (Speranza et al., 2012). Increased levels of ROS lead to the activation of the antioxidant system, possibly through a direct effect of ROS on signaling pathways (Rodriguez Milla et al., 2003; Richards et al., 2015). One gene (POPTRDRAFT_822482) involved in peroxidase activity was detected from clustering analysis. Peoxidases are a group of enzymes that protect cells against oxidative damage. The other gene (POPTRDRAFT_563887) involved in ATP-binding was also detected (Figure 4). These types of highly dynamic genes might play key roles in PG. Wang et al. (2008) reported that rescue- and transcription-related genes are overrepresented or markedly up-regulated during PG in *Arabidopsis*. The GO categories related to transport (Go biologycal process) and protein complex (Go cellular component) were both most significantly enriched in PG. The categories associated with transporter activities and macromolecular complex were reported to be the most over-represented in mature pollen compared with pollen tubes (Hafidh et al., 2012). The enrichments of GO categories may provide important hints about pollen biology.

PTG had a distinct gene function distribution from PG. The percentage of up-regulated genes involved in catalysis, enzyme regulation, molecular transduction, translation regulation, nutrient reservoir, and transporter activity was increased during PTG, especially those in the categories of molecular transducer, translation regulator, and nutrient reservoir. Moreover, the three types of genes were down-regulated during PG (Figure 3, Supplementary Table S7). These findings agree with the two major functional categories found to be up-regulated in *Arabidopsis* during PTG, signaling and transporter-related activity (Wang et al., 2008). Molecular transducer- and transporter-related genes are involved in the regulation of the polarized tip growth of PTs (Hepler et al., 2001). This process is regulated by complex signal crosstalk and requires a large number of transporters to supply sufficient materials for the rapid growth of PTs (Hepler et al., 2001; Holdaway-Clarke and Hepler, 2003). The categories or subcategories related to nutrient reservoir activity, translation, protein transport, and turnover and signal transduction are highly over-represented in the subset of 104 pollen-tube/root-hair overlapping genes (Hafidh et al., 2012). In particular, genes associated with chromatin remodeling and transcription, signal transduction, and transport previously are over-represented in pollen tubes that has grown semi-*in vivo* through the pistil and can thus mediate pollen-tube growth and guidance through the style (Qin et al., 2009). Nine genes identified from clustering analysis are significantly up-regulated during PTG but significantly down-regulated during PG (Figure 4). Four genes are involved in protein kinase activity, microtubule motor activity, catalytic activity, and protein ubiquitination. Ubiquitin-mediated proteolysis is reportedly involved in spermatogenesis in *Arabidopsis* (Borges et al., 2008). The molecular functions of the other five genes remain unknown. The molecular functions of these four characterized genes are very consistent with the physiological characteristics of PTG and spermatogenesis.

In addition, clustering analysis also showed that some genes were up-regulated during both PG and PTG (Figure 4), including genes involved in pectinesterase activity, membrane or transport activity, regulation of transcription, protein nuclear import, protein modification, and calcium ion binding. These functions are closely associated with polarized PTG (Pina et al., 2005; Wang et al., 2008; Qin et al., 2009). The functions of more than half of the genes that were up-regulated during PG and PTG remain unknown.

### Pathways closely associated with PG and PTG

The MAPK signaling pathway, regulation of the actin cytoskeleton, focal adhesion, GnRH signaling, and chemokine signaling were only significantly enriched in PG (Table 3, Supplementary Table S9). MAPK cascades are essential for many signaling pathways in plants, and there is often crosstalk between members of different signaling pathways (Mishra et al., 2006). We identified encode classical MAPK pathway–related proteins such as CACN, PKC, MEK1, MEK2, ERK. Genes encoding MEKK1, PAK1/2, MEKK2/3, MUK, MLTK, and TAK1 were found to be significantly down-regulated, whereas genes encoding HSP72 and PPP3C were significantly up-regulated in the pathway. CACN-1 was reported that it was required cell-autonomously to control distal tip cells migration (Tannoury et al., 2010). Wang et al. (2008) proposed that Hsps may function as molecular chaperones (for review, see Miernyk, 1999) involved in the regulation of protein modification during PG and PTG, which is associated with the rapid protein synthesis and high physiological activity in germinating pollen and pollen tubes. Here there is crosstalk among GnRH signaling, focal adhesion, the MAPK signaling pathway. There is also crosstalk between regulation of the actin cytoskeleton and focal adhesion. These crosstalks were mainly linked by genes encoding PAK, MEK, and ERK. They were found to be significantly down-regulated in above pathways (Supplementary Figures S3–S7). The common function of PAK is the activation of MAPK cascades and the regulation of cytoskeletal structure through effects on the actin and tubulin cytoskeletons (Hofmann et al., 2004). ERK1,2 activation has also been shown to be required for cell proliferation and transformation in mice (Troppmair et al., 1994). It is well known that the dynamic organization of the actin cytoskeleton plays a fundamental role in PG and PTG. Genes encoding proteins involved in the regulation of the actin cytoskeleton, such as GIT1, PI4P5K, mDia, and gelsolin (GSN), were significantly down-regulated during PG (Supplementary Figure S4). GIT1 is a multidomain protein that is thought to function as an integrator of signaling pathways controlling vesicle trafficking, adhesion, and cytoskeletal organization (Manabe et al., 2002). The poppy (Papaver rhoeas) GSN, in addition to Ca^2+^-regulated severing, can nucleate new actin filaments during assembly and also cap the barbed end of actin filaments (Huang et al., 2004). These five pathways were also closely related to PG.

Different pathways were significantly enriched during PTG and sperm cell formation compared with PG. Some metabolic and signaling pathways, such as sphingolipid metabolism, galactose metabolism, glycolysis, glycerophospholipid metabolism, oxidative phosphorylation, and calcium signaling, provide large quantities of material and energy for the extremely rapid PTG (Table 4, Supplementary Table S10). The vesicle transport–associated SNARE pathway, which is presumably required for material transport during PTG, was also enriched in PTG. Another significantly enriched pathway, ubiquitin-mediated proteolysis (Table 4), plays an essential role in male gametogenesis in mice and humans (Baarends et al., 1999a,b). Ubiquitin-mediated proteolysis is also one of the overrepresented GO categories in *Arabidopsis* sperm cells (Borges et al., 2008). The majority of the 25 known genes that encode ubiquitin pathway–related proteins, such as ubiquitin-conjugating enzyme and ubiquitin ligase, were up-regulated in the PTG samples. For example, four genes encoding E2-related proteins (UBE2I, UBE2J1, UBE2N, and UBE2W) and three genes encoding HECT-type E3-related proteins (NEDD4, HERC2, and HERC3) were up-regulated. Genes encoding the multisubunit RING-finger-type E3-related proteins (Skip1, F-box, DDB1, and DCAF) were all significantly differential expressed during PTG (Supplementary Figure S8). Skp1/Cullin1/F-box complexes are involved in the ubiquitination of proteins targeted for proteasome-dependent degradation and are also known to control the cell cycle and diversified developmental processes (Cardozo and Pagano, 2004; Lechner et al., 2006; Hafidh et al., 2012). The Cullin4/DDB1/DCAF complex may play important roles in multiple developmental processes including plant embryogenesis (Zhang et al., 2008). These pathways, other than sphingolipid metabolism, were only significantly enriched in PTG. There is also crosstalk among galactose metabolism, glycerophospholipid metabolism and glycolysis.

The pathway enrichment analysis revealed that 12 biological pathways were significantly enriched in both PG and PTG (Tables 3, 4; Supplementary Tables S9, 10), even though the expression levels of some of the genes within those pathways showed differences in PG and PTG. Although the role of some pathways or genes in pollen germination and PTG is not clear. DEGs in the above pathways will be the attractive candidates for further analysis.

## Author contributions

CY designed experiment. LZ carried out experiments, analyzed experimental results, and wrote the manuscript. HY assisted with results analysis. WG assisted with organizing figures and tables.

### Conflict of interest statement

The authors declare that the research was conducted in the absence of any commercial or financial relationships that could be construed as a potential conflict of interest.

## Supplementary material

Supplementary Table S1 **Genes and primer sequences selected for qRT-PCR**.

Supplementary Table S2 **Summary of Illumina/Solexa clean tags mapping to sense or antisense genes**.

Supplementary Table S3 **Summary of sense or antisense-strand transcripts (these genes are all unigenes)**.

Supplementary Table S4 **Total genes expressed in at least one of MP, HP, and PT**.

Supplementary Table S5 **Genes expressed in the three stages**.

Supplementary Table S6 **DEGs during PG and PTG**.

Supplementary Table S7 **DEGs classified into 10 functional categories during PG**.

Supplementary Table S8 **Significantly enriched GO terms and DEGs in the terms of PG and PTG**.

Supplementary Table S9 **Enriched pathways and DEGs in the pathways of MPvsHP**.

Supplementary Table S10 **Enriched pathways and DEGs in the pathways of HPvsPT**.

Supplementary Figure S1 **Saturation evaluation of differential expression in the three samples**. When sequencing reached ≥2, 1.5, and 1.0 million reads for MP, HP, and PT, respectively, the number of newly detected genes essentially stopped increasing.

Supplementary Figure S2 **Distribution of fold-change in tag number among the three libraries**.

Supplementary Figure S3 **Overview of the MAPK signaling pathway taken from KEGG database—one of the pathways that was significantly enriched during PG (proteins outlined in red were up-regulated, and proteins outlined in green were down-regulated)**.

Supplementary Figure S4 **Overview of actin cytoskeleton regulation pathway taken from KEGG database—one of the pathways that was significantly enriched during PG (proteins outlined in green were down-regulated)**.

Supplementary Figure S5 **Overview of focal adhension pathway taken from KEGG database—one of the pathways that was significantly enriched during PG (proteins outlined in green were down-regulated)**.

Supplementary Figure S6 **Overview of GnRH signaling pathway taken from KEGG database—one of the pathways that was significantly enriched during PG (proteins outlined in green were down-regulated)**.

Supplementary Figure S7 **Overview of chemokine signaling pathway taken from KEGG database—one of the pathways that was significantly enriched during PG (proteins outlined in green were down-regulated)**.

Supplementary Figure S8 **Overview of the ubiquitin (Ub)-mediated proteolysis pathway taken from KEGG database—one of the pathways that was significantly enriched during PTG (proteins outlined in red were up-regulated, and proteins outlined in green were down-regulated)**.

## References

[B1] Audic S. Claverie J. M. (1997). The significance of digital gene expression profiles. Genome Res. 7, 986–995. 9331369 10.1101/gr.7.10.986

[B2] Azad A. K. M. Lawen A. Keith J. M. (2015). Prediction of signaling cross-talks contributing to acquired drug resistance in breast cancer cells by Bayesian statistical modeling. BMC Syst. Biol. 2:9 10.1186/s12918-014-0135-x PMC4307189 25599599

[B3] Baarends W. M. Hoogerbrugge J. W. Roest H. P. Oom M. Vreeburg J. Hoeijimakers J. H. J. (1999a). Histone ubiquitination and chromatin remodeling in mouse spermatogenesis. Dev. Biol. 207, 322–333. 10.1006/dbio.1998.9155 10068466

[B4] Baarends W. M. Roest H. P. Grootegoed J. A. (1999b). The ubiquitin system in gametogenesis. Mol. Cell. Endocrinol. 25, 5–16. 10.1016/S0303-7207(99)00060-X 10411315

[B5] Becker J. D. Boavida L. C. Carneiro J. Haury M. Feijó J. A. (2003). Transcriptional profiling of Arabidopsis tissues reveals the unique characteristics of the pollen transcriptome. Plant Physiol. 133, 713–725. 10.1104/pp.103.028241 14500793 PMC219046

[B6] Benjamini Y. Yekutieli D. (2001). The control of the false discovery rate in multiple testing under dependency. Ann. Stat. 29, 1165–1188.

[B7] Borges F. Gomes G. Gardner R. Moreno N. McCormick S. Feijó J. A.. (2008). Comparative transcriptomics of Arabidopsis sperm cells. Plant Physiol. 148, 1168–1181. 10.1104/pp.108.125229 18667720 PMC2556834

[B8] Cardozo T. Pagano M. (2004). The SCF ubquitin ligase: insights into a molecular machine. Nat. Rev. Mol. Cell Biol. 5, 739–751. 10.1038/nrm1471 15340381

[B9] Eisen M. B. Spellman P. T. Brown P. O. Botstein D. (1998). Cluster analysis and display of genome-wide expression patterns. Proc. Natl. Acad. Sci. U.S.A. 95, 14863–14868. 10.1073/pnas.95.25.14863 9843981 PMC24541

[B10] Feijó J. A. Costa S. S. Prado A. M. Becker J. D. Certal A. C. (2004). Signalling by tips. Curr. Opin. Plant Biol. 7, 589–598. 10.1016/j.pbi.2004.07.014 15337103

[B11] Feijó J. A. Sainhas J. Holdaway-Clarke T. Cordeiro M. S. Kunkel J. G. Hepler P. K. (2001). Cellular oscillations and the regulation of growth: the pollen tube paradigm. Bioessays 23, 86–94. 10.1002/1521-1878(200101)23:1<86::AID-BIES1011>3.0.CO;2-D 11135313

[B12] Guyon V. N. Astwood J. D. Garner E. C. Dunker A. K. Taylor L. P. (2000). Isolation and characterization of cDNAs expressed in the early stages of flavonol-induced pollen germination in petunia. Plant Physiol. 123, 699–710. 10.1104/pp.123.2.699 10859200 PMC59038

[B13] Haerizadeh F. Wong C. Bhalla P. Gresshoff P. Singh M. (2009). Genomic expression profiling of mature soybean (*Glycine max*) pollen. BMC Plant Biol. 9:25. 10.1186/1471-2229-9-25 19265555 PMC2660330

[B14] Hafidh S. Breznenová K. RùŽièka P. Feciková J. Èapková V. Honys D. (2012). Comprehensive analysis of tobacco pollen transcriptome unveils common pathways in polar cell expansion and underlying heterochronic shift during spermatogenesis. BMC Plant Biol. 12:24. 10.1186/1471-2229-12-24 22340370 PMC3305590

[B15] Hao H. Li Y. Hu Y. Lin J. (2005). Inhibition of RNA and protein synthesis in pollen tube development of pinus bungeana by actinomycin D and cycloheximide. New Phytol. 165, 721–729. 10.1111/j.1469-8137.2004.01290.x 15720683

[B16] Hepler P. K. Vidali L. Cheung A. Y. (2001). Polarized cell growth in higher plants. Annu. Rev. Cell Dev. Biol. 17, 159–187. 10.1146/annurev.cellbio.17.1.159 11687487

[B17] t Hoen P. A. C. Ariyurek Y. Thygesen H. H. Vreugdenhil E. Vossen R. H. A. M. de Menezes R. X.. (2008). Deep sequencing -based expression analysis shows major advances in robustness, resolution and inter-lab portability over five microarray platforms. Nucleic Acids Res. 36:e141. 10.1093/nar/gkn705 18927111 PMC2588528

[B18] Hofmann C. Shepelev M. Chernoff J. (2004). The genetics of Pak. J. Cell Sci. 117, 4343–4354. 10.1242/jcs.01392 15331659

[B19] Holdaway-Clarke T. L. Hepler P. K. (2003). Control of pollen tube growth: role of ion gradients and fluxes. New Phytol. 159, 539–563. 10.1046/j.1469-8137.2003.00847.x 33873604

[B20] Honys D. Twell D. (2003). Comparative analysis of the Arabidopsis pollen transcriptome. Plant Physiol. 132, 640–652. 10.1104/pp.103.020925 12805594 PMC167004

[B21] Honys D. Twell D. (2004). Transcriptome analysis of haploid male gametophyte development in Arabidopsis. Genome Biol. 5:R85. 10.1186/gb-2004-5-11-r85 15535861 PMC545776

[B22] Huang S. Blanchoin L. Chaudhry F. Franklin-Tong V. E. Staiger C. J. (2004). A gelsolin-like protein from Papaver rhoeas pollen (PrABP80) stimulates calcium-regulated severing and depolymerization of actin filaments. J. Biol. Chem. 279, 23364–23375. 10.1074/jbc.M312973200 15039433

[B23] Lechner E. Achard P. Vansiri A. Potuschak T. Genschik P. (2006). F-box proteins everywhere. Curr. Opin. Plant Biol. 9, 631–638. 10.1016/j.pbi.2006.09.003 17005440

[B24] Lee J. Y. Lee D. H. (2003). Use of serial analysis of gene expression technology to reveal changes in gene expression in Arabidopsis pollen undergoing cold stress. Plant Physiol. 132, 517–529. 10.1104/pp.103.020511 12805584 PMC166994

[B25] Lin S. Y. Chen P. W. Chuang M. H. Juntawong P. Bailey-Serres J. Jauh G. Y. (2014). Profiling of translatomes of *in vivo*–grown pollen tubes reveals genes with roles in micropylar guidance during pollination in Arabidopsis. Plant Cell 26, 602–618. 10.1105/tpc.113.121335 24532595 PMC3967028

[B26] Livak K. J. Schmittgen T. D. (2001). Analysis of relative gene expression data using real-time quantitative PCR and the 2(-Delta Delta C(T)) Method. Methods 25, 402–408. 10.1006/meth.2001.1262 11846609

[B27] Manabe R. Kovalenko M. Webb D. J. Horwitz A. R. (2002). GIT1 functions in a motile, multi-molecular signaling complex that regulates protrusive activity and cell migration. J. Cell Sci. 115, 1497–1510. 11896197 10.1242/jcs.115.7.1497

[B28] Mascarenhas J. P. (1975). The biochemistry of angiosperm pollen development. Bot. Rev. 41, 259–314. 10.1007/BF02860839

[B29] Mascarenhas J. P. (1993). Molecular mechanisms of pollen tube growth and differentiation. Plant Cell 5, 1303–1314. 10.1105/tpc.5.10.1303 12271030 PMC160363

[B30] Mascarenhas J. P. Mermelstein J. (1981). Messenger RNAs: their utilization and degradation during pollen germination and tube growth. Acta Soc. Bot. Pol. 50, 13–20. 10.5586/asbp.1981.002

[B31] McCormick S. (2004). Control of male gametophyte development. Plant Cell 16, S142–S153. 10.1105/tpc.016659 15037731 PMC2643393

[B32] Miernyk J. A. (1999). Protein folding in the plant cell. Plant Physiol. 121, 695–703. 10.1104/pp.121.3.695 10557217 PMC1539232

[B33] Mishra G. Zhang W. H. Deng F. Zhao J. Wang X. M. (2006). A bifurcating pathway directs abscisic acid effects on stomatal closure and opening in Arabidopsis. Science 312, 264–266. 10.1126/science.1123769 16614222

[B34] Modjtahedi H. Cho B. C. Michel M. C. Solca F. (2014). A comprehensive review of the preclinical efficacy profile of the ErbB family blocker afatinib in cancer. Naunyn Schmiedebergs Arch Pharmacol. 387, 505–521. 10.1007/s00210-014-0967-3 24643470 PMC4019832

[B35] Morrissy A. S. Morin R. D. Delaney A. Zeng T. McDonald H. Jones S. J.. (2009). Next-generation tag sequencing for cancer gene expression profiling. Genome Res. 19, 1825–1835. 10.1101/gr.094482.109 19541910 PMC2765282

[B36] Pina C. Pinto F. Feijó J. A. Becker J. D. (2005). Gene family analysis of the Arabidopsis pollen transcriptome reveals biological implications for cell growth, division control, and gene expression regulation. Plant Physiol. 138, 744–756. 10.1104/pp.104.057935 15908605 PMC1150393

[B37] Qin Y. Leydon A. R. Manziello A. Pandey R. Mount D. Denic S.. (2009). Penetration of the stigma and style elicits a novel transcriptome in pollen tubes, pointing to genes critical for growth in a pistil. PLoS Genet. 5:e1000621. 10.1371/journal.pgen.1000621 19714218 PMC2726614

[B38] Richards S. L. Wilkins K. A. Swarbreck S. M. Anderson A. A. Habib N. Smith A. G.. (2015). The hydroxyl radical in plants: from seed to seed. J. Exp. Bot. 66, 37–46. 10.1093/jxb/eru398 25294918

[B39] Rodriguez Milla M. A. Maurer A. Rodriguez Huete A. Gustafson J. P. (2003). Glutathione peroxidase genes in Arabidopsis are ubiquitous and regulated by abiotic stresses through diverse signaling pathways. Plant J. 36, 602–615. 10.1046/j.1365-313X.2003.01901.x 14617062

[B40] Saldanha A. J. (2004). Java treeview–extensible visualization of microarray data. Bioinformatics 20, 3246–3248. 10.1093/bioinformatics/bth349 15180930

[B41] Singh M. B. Bhalla P. L. (2007). Control of male germ-cell development in flowering plants. Bioessays 29, 1124–1132. 10.1002/bies.20660 17935220

[B42] Speranza A. Crinelli R. Scoccianti V. Geitmann A. (2012). Reactive oxygen species are involved in pollen tube initiation in kiwifruit. Plant Biol. (Stuttg). 4, 64–76. 10.1111/j.1438-8677.2011.00479.x 21973108

[B43] Sun Q. Zhou G. F. Cai Y. F. Fan Y. H. Zhu X. Y. Liu Y.. (2012). Transcriptome analysis of stem development in the tumourous stem mustard Brassica juncea var. tumida Tsen et Lee by RNA sequencing. BMC Plant Biol. 12:53. 10.1186/1471-2229-12-53 22520079 PMC3349559

[B44] Tannoury H. Rodriguez V. Kovacevic I. Ibourk M. Lee M. Cram E. J. (2010). CACN-1/Cactin interacts genetically with MIG-2 GTPase signaling to control distal tip cell migration in C. elegans. Dev. Biol. 341, 176–185. 10.1016/j.ydbio.2010.02.025 20188721 PMC2854247

[B45] Troppmair J. Bruder J. T. Munoz H. Lloyd P. A. Kyriakis J. Banerjee P.. (1994). Mitogen-activated protein kinase/extracellular signal-regulated protein kinase activation by oncogenes, serum, and 12-O-tetradecanoylphorbol-13-acetate requires Raf and is necessary for transformation. J. Biol. Chem. 269, 7030–7035. 8120067

[B46] Troppmair J. Bruder J. T. Munoz H. Lloyd P. A. Kyriakis J. Banerjee P. (2002). Pollen developmental biology, in Plant Reproduction, Annual Plant Reviews, Vol. 6, eds O'Neill S. D. Roberts J. A. (Sheffield: Sheffield Academic Press), 83–135.

[B47] Twell D. Oh S. A. Honys D. (2006). Pollen development, a genetic and transcriptomic view. Plant Cell Monogr. 3, 15–45. 10.1007/7089_042

[B48] Wang M. L. Hsu C. M. Chang L. C. Wang C. S. Su T. H. Huang Y. J. (2004). Gene expression profiles of cold-stored and fresh pollen to investigate pollen germination and growth. Plant Cell Physiol. 45, 1519–1528. 10.1093/pcp/pch174 15564535

[B49] Wang Y. Zhang W. Z. Song L. F. Zou J. J. Su Z. Wu W. H. (2008). Transcriptome analyses show changes in gene expression to accompany pollen germination and tube growth in Arabidopsis. Plant Physiol. 148, 1201–1211. 10.1104/pp.108.126375 18775970 PMC2577266

[B50] Wei L. Q. Xu W. Y. Deng Z. Y. Su Z. Xue Y. B. Wang T. (2010). Genome-scale analysis and comparison of gene expression profiles in developing and germinated pollen in *Oryza sativa*. BMC Genomics 11:338. 10.1186/1471-2164-11-338 20507633 PMC2895629

[B51] Wittink F. R. A. Knuiman B. Derksen J. Capkova V. Twell D. Schrauwen J. A. M. (2000). Immunochemical identification of gelsolin by western blotting in maize pollen. Chinese Sci. Bull. 45, 256–258. 10.1007/BF02884686

[B52] Zhang Y. Feng S. H. Chen F. F. Chen H. D. Wang J. McCall C.. (2008). Arabidopsis DDB1-Cul4 associated factor1 forms a nuclear E3 ubiquitin ligase with DDB1 and Cul4 that is involved in multiple plant developmental processes. Plant Cell 20, 1437–1455. 10.1105/tpc.108.058891 18552200 PMC2483375

[B53] Zimmermann P. Hirsch-Hoffmann M. Henning L. Gruissem W. (2004). Genevestigator. Arabidopsis microarray database and analysis toolbox. Plant Physiol. 136, 2621–2632. 10.1104/pp.104.046367 15375207 PMC523327

