# Corrigendum: Digital Gene Expression Analysis of *Populus simonii* × *P. nigra* Pollen Germination and Tube Growth

**DOI:** 10.3389/fpls.2016.01056

**Published:** 2016-07-19

**Authors:** Li-Juan Zhao, Hong-Mei Yuan, Wen-Dong Guo, Chuan-Ping Yang

**Affiliations:** ^1^State Key Laboratory of Tree Genetics and Breeding, Northeast Forestry UniversityHarbin, China; ^2^Department of Crop Molecular Breeding, Crop Breeding Institute, Heilongjiang Academy of Agricultural SciencesHarbin, China; ^3^Medical Plant Research Center, Economic Crop Institute, Heilongjiang Academy of Agricultural SciencesHarbin, China; ^4^Biotechnology Research Center, Institute of Natural Resources and Ecology, Heilongjiang Academy of SciencesHarbin, China

**Keywords:** *Populus simonii* × *P. nigra* pollen, pollen germination, pollen tube growth, transcription, DGE, differentially expressed genes

Reason for Corrigendum:

There was a mistake in the color of the arrow of Figure 1A as published. Red arrows should be white and white arrows should be red in Figure 1A. The corrected Figure 1 is below. The authors apologize for the mistake. This error does not change the scientific conclusions of the article in any way.

**Figure 1:**
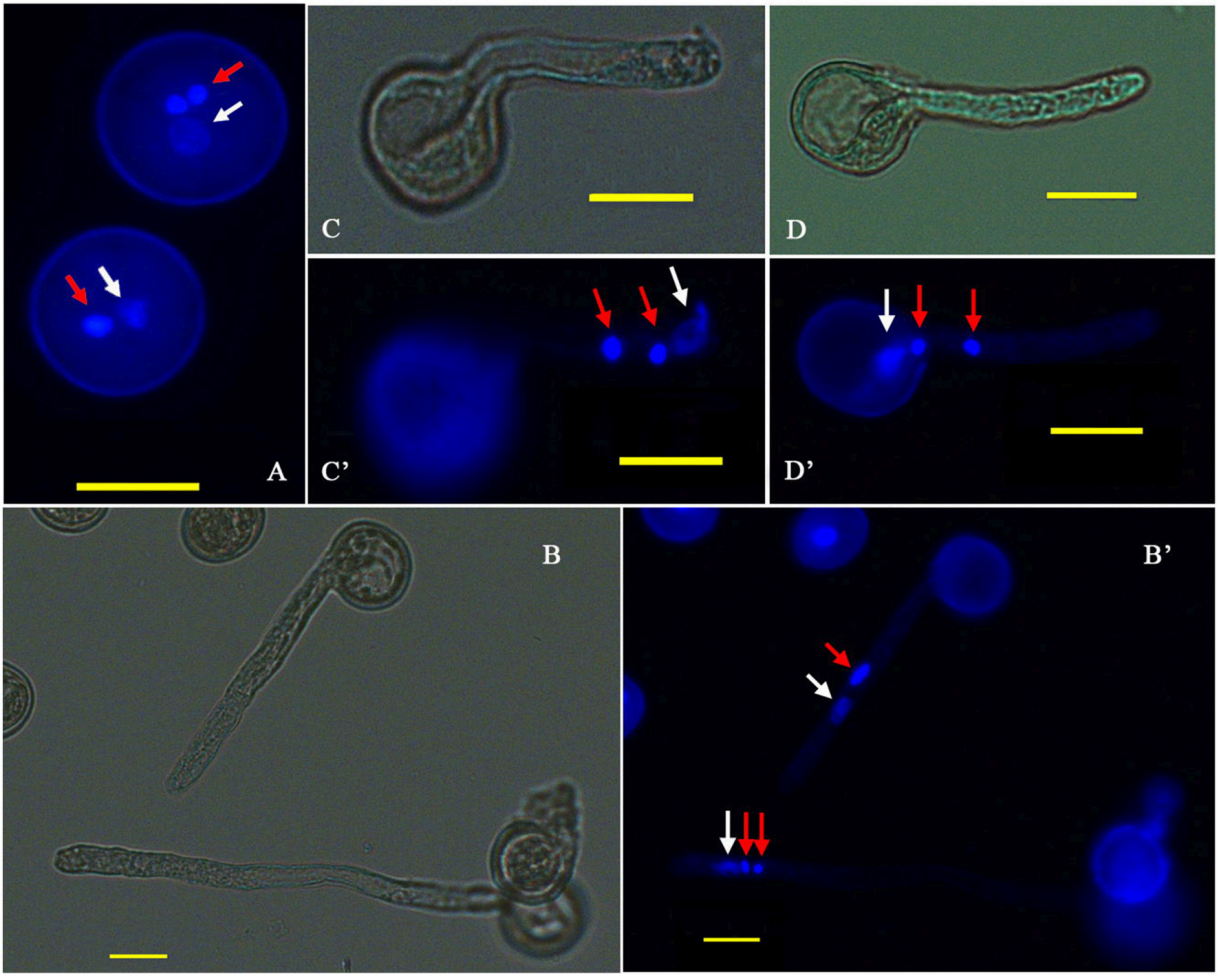
**Cytological observation of *P. simonii* × *P. nigra* PG and PTG. (A)** DAPI fluorescence image of two-celled and three-celled pollen. **(B,B**′**)** Bright-field and DAPI fluorescence images of the two and three nuclei states in the tube; red arrows show generative nuclei or two sperm nuclei, and white arrows show vegetative nuclei. **(C,C**′**)** Bright-field and DAPI fluorescence images, respectively, of the vegetative nucleus leading the sperm nucleus into the PT. **(D,D**′**)** Bright-field and DAPI fluorescence images, respectively, of two sperm nuclei moving into the tube before the vegetative nucleus.

## Author contributions

CY designed experiment. LZ carried out experiments, analyzed experimental results, and wrote the manuscript. HY assisted with results analysis. WG assisted with organizing figures and tables.

### Conflict of interest statement

The authors declare that the research was conducted in the absence of any commercial or financial relationships that could be construed as a potential conflict of interest.

