# Supplementary material for: Digital Gene Expression Analysis of Populus simonii × P. nigra Pollen Germination and Tube Growth
Source: Front Plant Sci. 2016 Jun 15;7:825. doi: 10.3389/fpls.2016.00825 (PMC4908133; doi:10.3389/fpls.2016.00825)
Supplement: Supplementary Table S1 — Genes and primer sequences selected for qRT-PCR. [file Table1.doc]

| **Supplementary Table 1.** Genes and primer sequences selected for qRT-PCR | | | |
| --- | --- | --- | --- |
| **Gene symbol** | **Description*** | **Primer sequences （5′- to -3′）** | **Amplicon size** |
| POPTRDRAFT_571733 | eugene3.00131097 | AAGAACACAGACAAGGCAGCA | 273 |
|  |  | TAAAGGAGAGAGCCAAGGAGC |  |
| POPTRDRAFT_549817 | eugene3.00012258 | GCTTCATAGACCTTCTCGCCT | 155 |
|  |  | CTCAACTTTTTCTTGCCTCCT |  |
| POPTRDRAFT_226184 | pseudo | TGTTGGTTCAGGAGGAGGTGT | 275 |
|  |  | ATATGCTGCATGCTCAGGGTG |  |
| POPTRDRAFT_822482 | estExt_fgenesh4_pg.C_LG_X1852 | TTGTCCTTTGTTCTTGCTCTCTC | 181 |
|  |  | TTTTGGATGTCACTCCTCTTTCT |  |
| POPTRDRAFT_551510 | eugene3.00020825 | GAGTGCCTCTTCCTTCCTGTA | 211 |
|  |  | GGCGATCAAAACCTTGTGTAT |  |
| POPTRDRAFT_653794 | grail3.0013010101 | CACCTCACTTTTCCTCCACCAC | 152 |
|  |  | CTCATCGCTTCCATCTTCTACC |  |
| POPTRDRAFT_758103 | fgenesh4_pg.C_LG_III001645 | TCAGATACAAGGCCCATCCT | 258 |
|  |  | TGCTTCAACTTTACCAGCGA |  |
| POPTRDRAFT_572572 | eugene3.00140617 | TGGCTAACCCAACAACACCTT | 173 |
|  |  | ACCCTCATTCACCTCCCTCTC |  |
| POPTRDRAFT_586725 | eugene3.02000004 | AAGTGGAGTCGGTGTTTGGT | 298 |
|  |  | GATTTGGATTGGGAAGGGTA |  |
| POPTRDRAFT_290778 | gw.535.12.1 | CCTCTTTTGGTCTTGACGGGT | 207 |
|  |  | TCTCTGATTTCGGGCTTGCTA |  |
| POPTRDRAFT_719293 | estExt_Genewise1_v1.C_LG_VII2998 | GAAGAGATTGAAGAGGGGATGG | 300 |
|  |  | AAGTTGGCTTGAGTTATATGGG |  |
| POPTRDRAFT_230571 | pseudo | AGCCTTGCTGTGTTCTTCTCC | 211 |
|  |  | CTTGCCTCCTATTGTGTTGGT |  |
| POPTRDRAFT_815726 | estExt_fgenesh4_pg.C_LG_I2723 | TGAGGAGATAATGGACGAAGG | 261 |
|  |  | GGGAGTTAACCCAGACAAGGC |  |

*****Referring*Populus trichocarpa* genome annotation
