# Supplementary material for: Digital Gene Expression Analysis of Populus simonii × P. nigra Pollen Germination and Tube Growth
Source: Front Plant Sci. 2016 Jun 15;7:825. doi: 10.3389/fpls.2016.00825 (PMC4908133; doi:10.3389/fpls.2016.00825)
Supplement: Supplementary Table S2 — Summary of Illumina/Solexa clean tags mapping to sense or antisense genes. [file Table2.doc]

| **Supplementary Table 2.** Summary of Illumina/Solexa clean tags mapping to sense or antisense genes | | | | | | | | | | | | |
| --- | --- | --- | --- | --- | --- | --- | --- | --- | --- | --- | --- | --- |
|  | MP | | | | HP | | | | PT | | | |
|  | Distinct Tags  Number % | | Total Tags Number % | | Distinct Tags  Number % | | Total Tags Number % | | Distinct Tags  Number % | | Total Tags Number % | |
| All tags mapping to sense genes | 53356 | 42.18 | 2285391 | 50.68 | 35186 | 35.60 | 1307175 | 43.72 | 35894 | 39.64 | 1183954 | 47.24 |
| All tags mapping to antisense genes | 5983 | 4.73 | 54815 | 1.22 | 6793 | 6.87 | 74385 | 2.49 | 3840 | 4.24 | 32030 | 1.28 |
| Unambiguous tags mapping to sense genes | 42692 | 33.75 | 1620150 | 35.93 | 27774 | 28.10 | 915750 | 30.63 | 28289 | 31.24 | 801057 | 31.96 |
| Unambiguous tags mapping to antisense genes | 4602 | 3.64 | 39504 | 0.88 | 5224 | 5.29 | 53242 | 1.78 | 2970 | 3.28 | 23473 | 0.94 |
