# Supplementary material for: Digital Gene Expression Analysis of Populus simonii × P. nigra Pollen Germination and Tube Growth
Source: Front Plant Sci. 2016 Jun 15;7:825. doi: 10.3389/fpls.2016.00825 (PMC4908133; doi:10.3389/fpls.2016.00825)

Supplementary Fig. 1 Saturation evaluation of different expression in three samples.
When sequencing amount reaches 2M, 1.5M and 1.0M or higher in MP, HP and PT respectively, the number of detected genes almost ceases to increase.

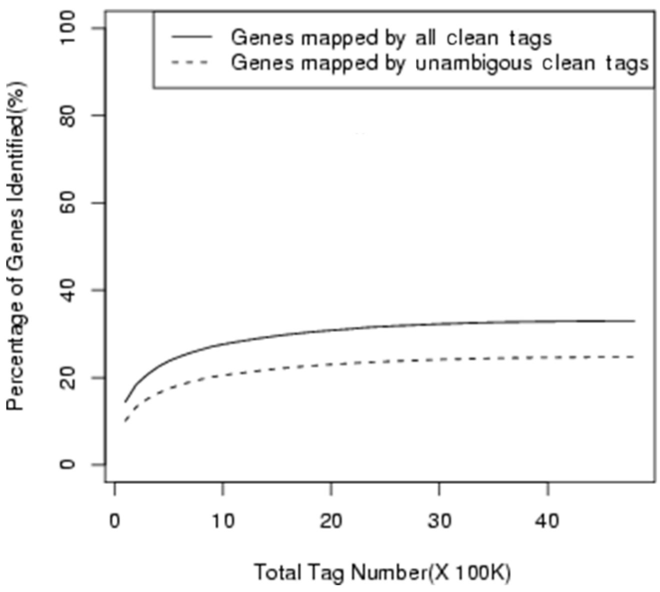

MP

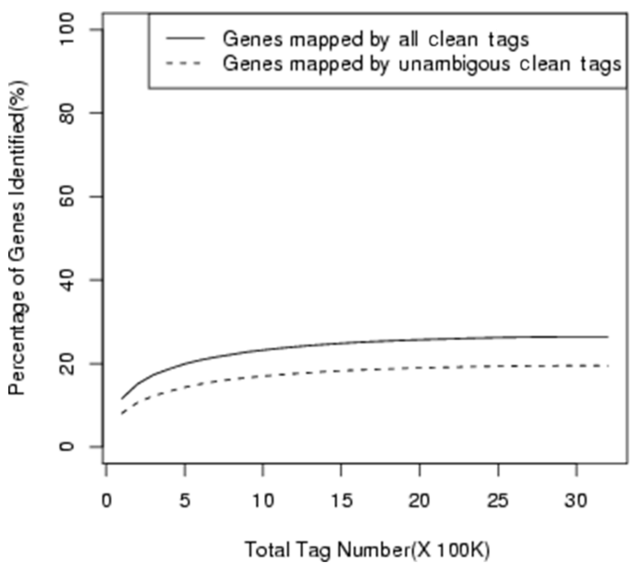

HP

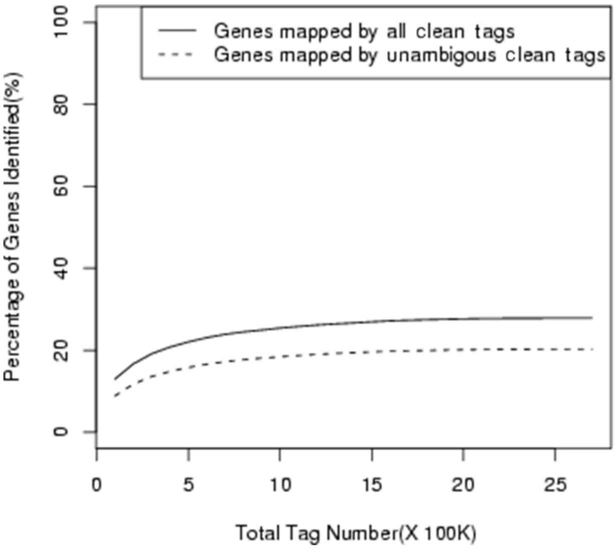
Genes mapped by all clean tags
Genes mapped by unambigous clean tags
Percentage of Genes Identified(%)
Total Tag Number(X 100K)
0
20
40
60
80
100
0
5
10
15
20
25

PT

Supplementary Fig. 2 The distribution of fold-changes in tag number among the three libraries.

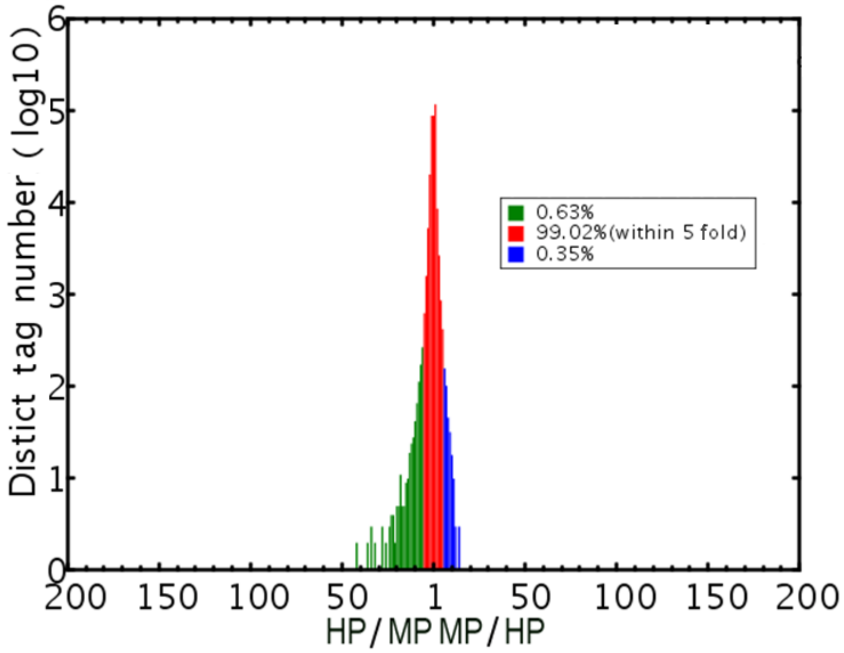

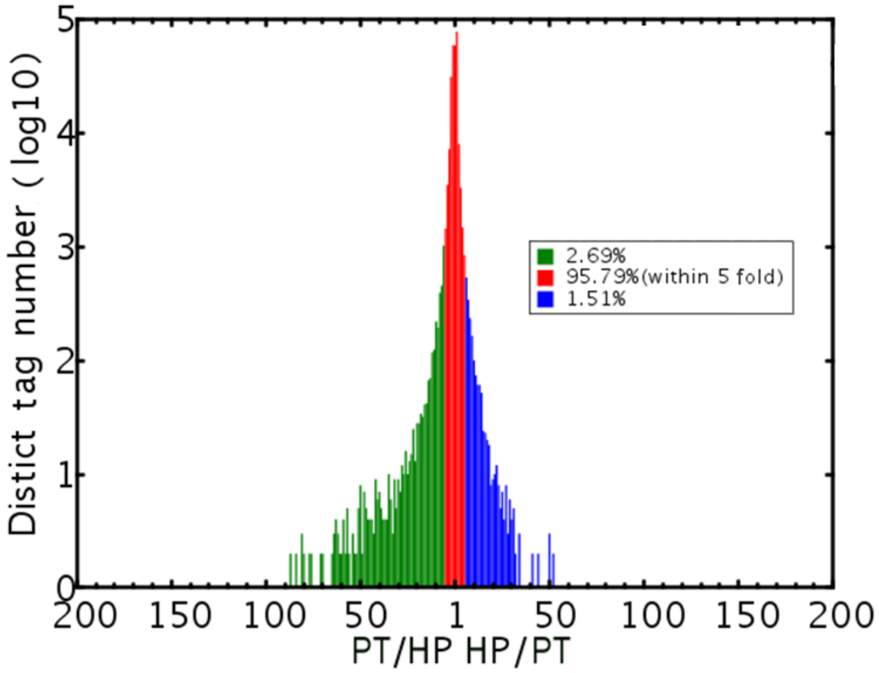

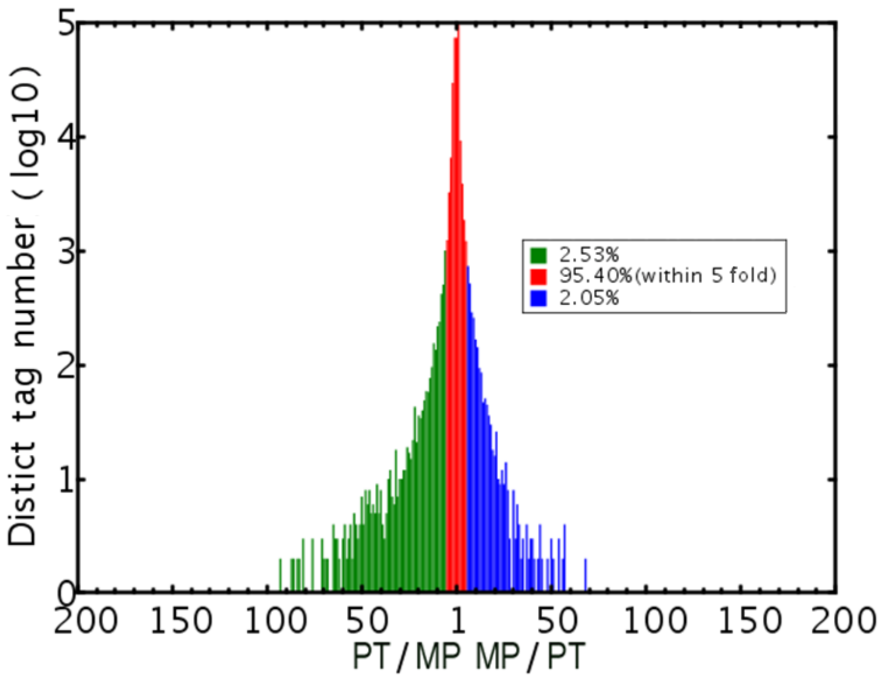

Distict tag number (log10)
0
1
2
3
4
5
200
150
100
50
1
50
100
150
200
PT / MP MP / PT
2.53%
95.40%(within 5 fold)
2.05%

Supplementary Fig. 3 One significantly enriched pathway during PG about MAPK signaling pathway (Red boxes represent up-regulated, Green boxes represent down-regulated). Taken from KEGG database.

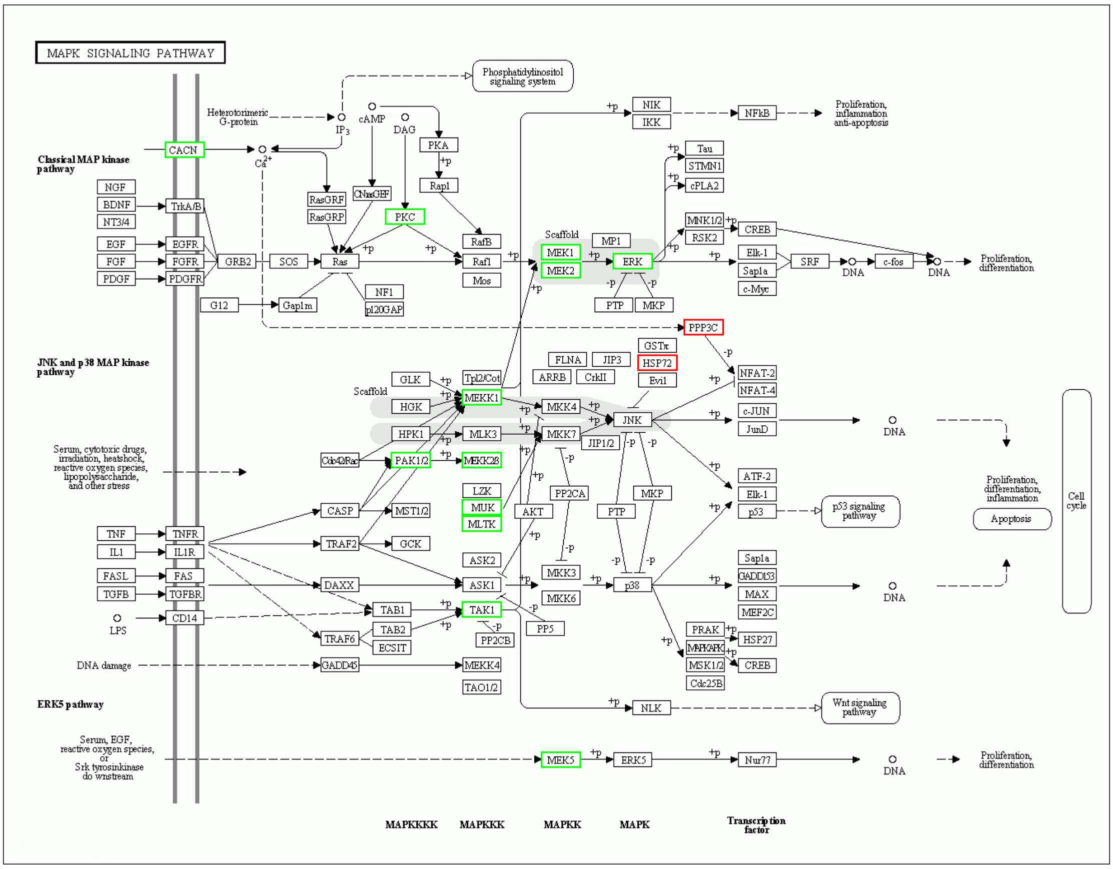

Supplementary Fig. 4 One significantly enriched pathway during PG about Regulation of actin cytoskeleton (Green boxes represent down-regulated). Taken from KEGG database.

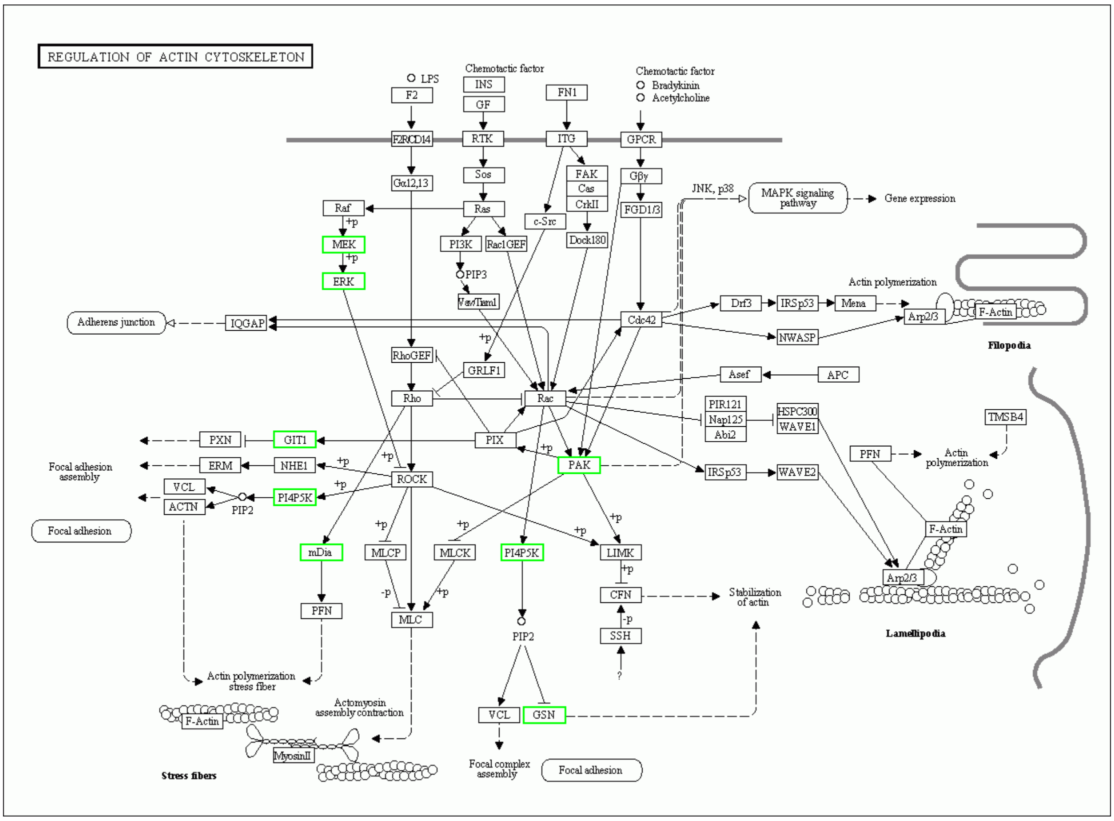

Supplementary Fig. 5 one significantly enriched pathway during PTG about focal adhesion (Red boxes represent up-regulated, Green boxes represent down-regulated). Taken from KEGG database.

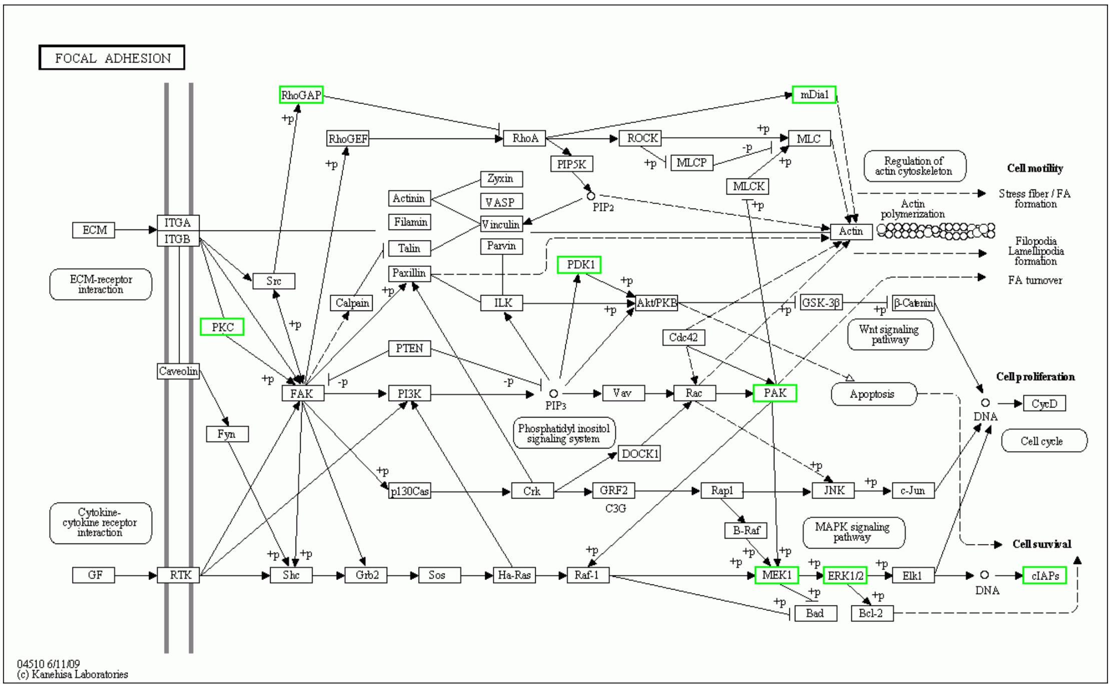

Supplementary Fig. 6 one significantly enriched pathway during PTG about GnRH signaling (Red boxes represent up-regulated, Green boxes represent down-regulated). Taken from KEGG database.

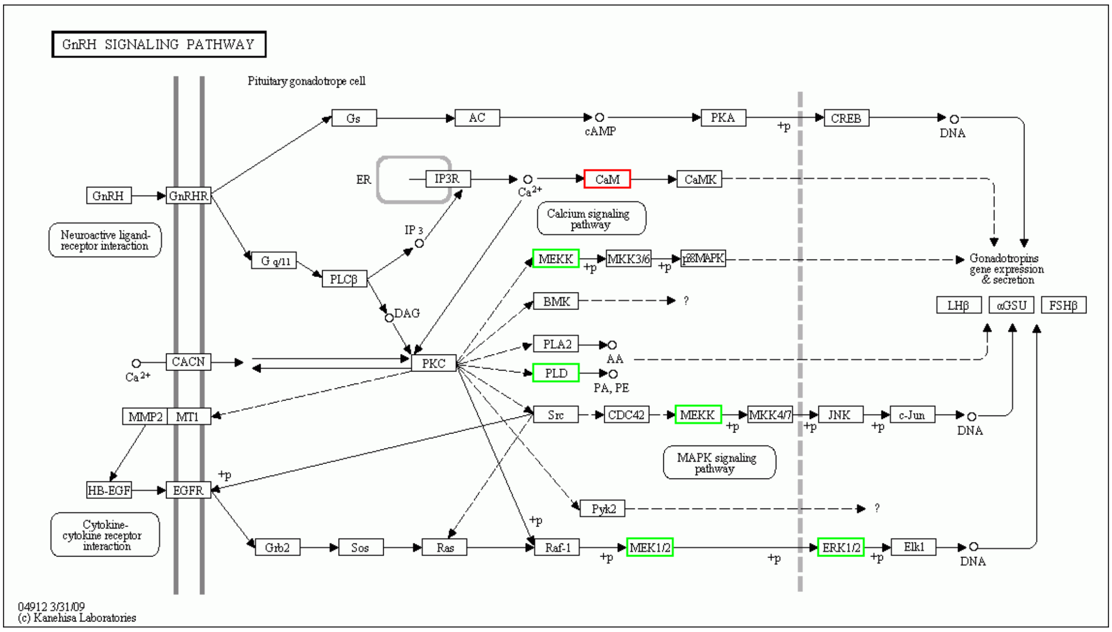

Supplementary Fig. 7 one significantly enriched pathway during PTG about chemokine signaling (Red boxes represent up-regulated, Green boxes represent down-regulated). Taken from KEGG database.

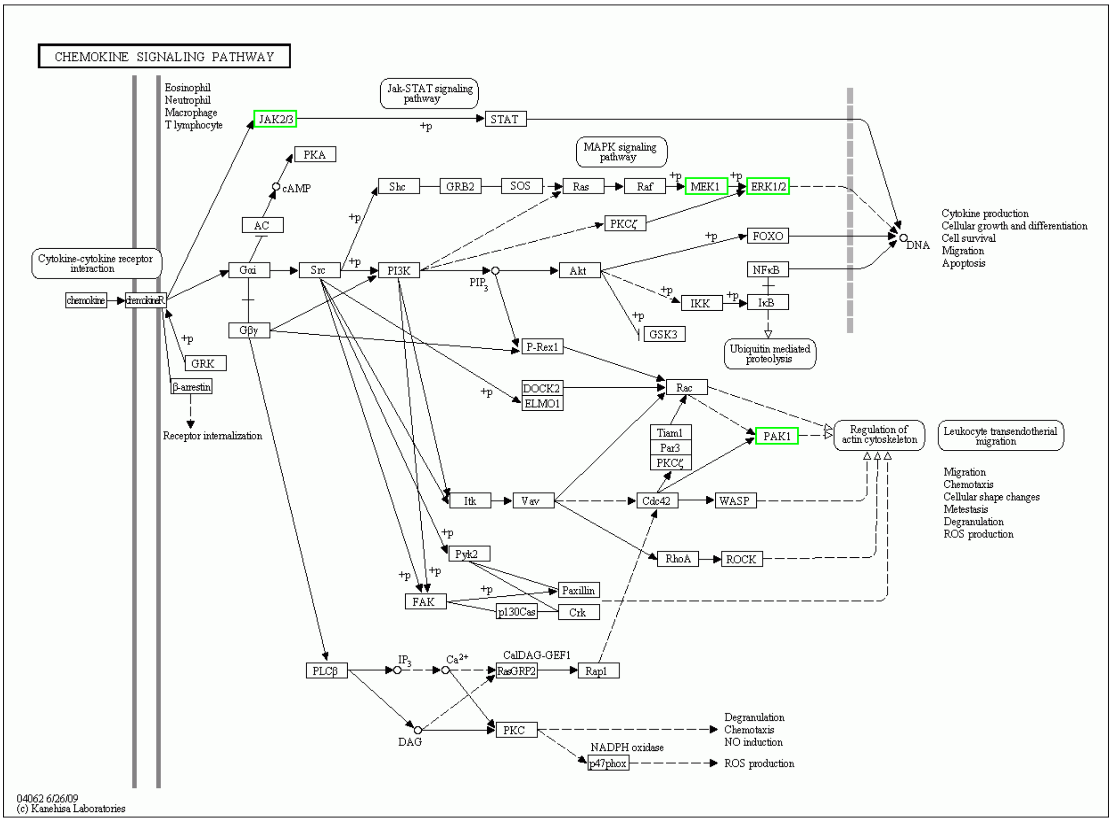

Supplementary Fig. 8 one significantly enriched pathway during PTG about Ubiquitin-mediated proteolysis (Red boxes represent up-regulated, Green boxes represent down-regulated). Taken from KEGG database.

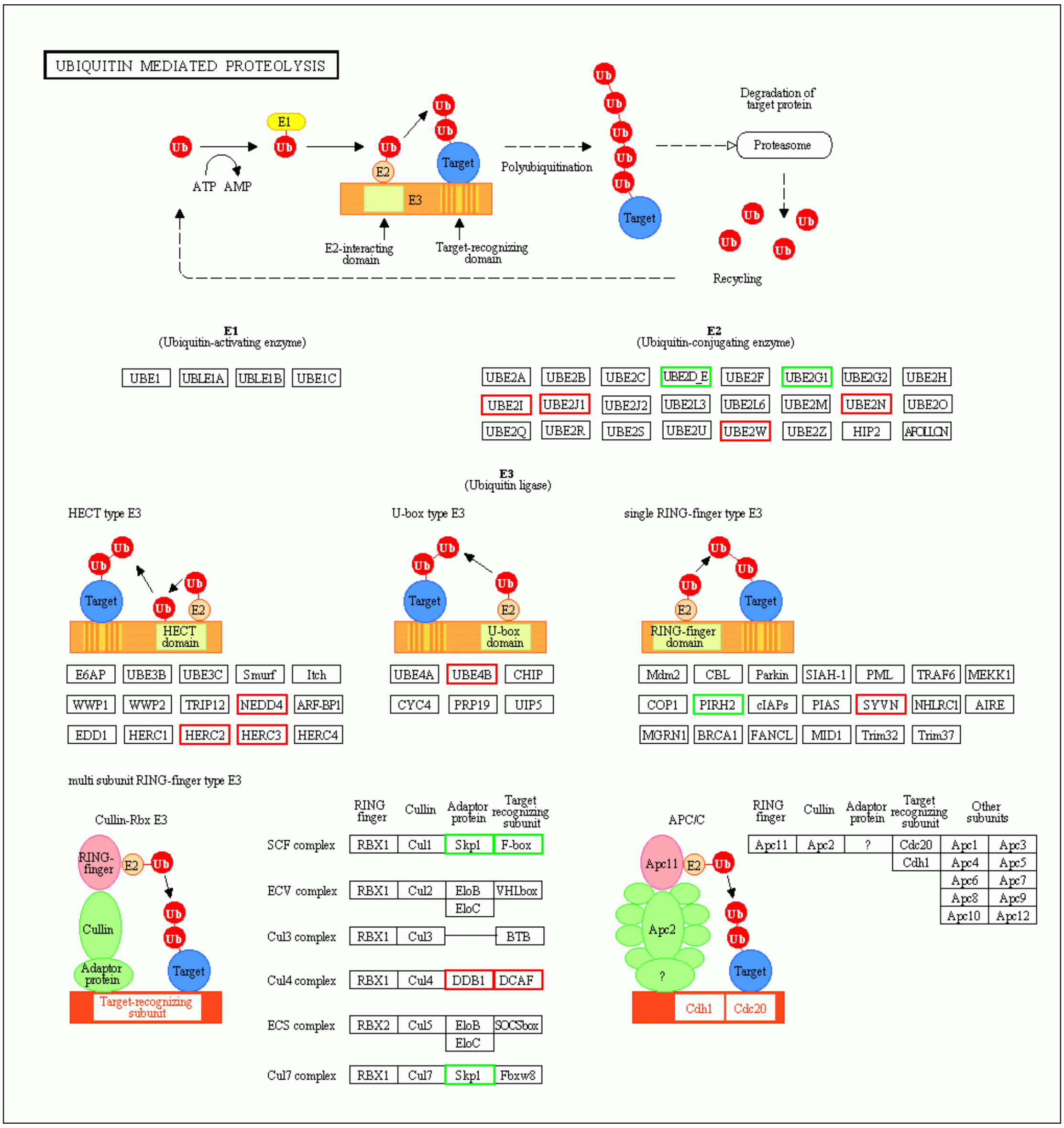

Supplement: Supplementary Figure S1 — Saturation evaluation of differential expression in the three samples. When sequencing reached ≥2, 1.5, and 1.0 million reads for MP, HP, and PT, respectively, the number of newly detected genes essentially stopped increasing. [file Presentation1.PDF]
